# Natural Product-Based Nanomedicine in the Treatment of Breast Cancer

**DOI:** 10.3390/pharmaceutics18091101

**Published:** 2026-09-01

**Authors:** Kaiyan Su, Yan Li, Dongmei Zhang, Yuxuan Zhou, Jianping Zhang, Hongyan Zhu, Yun Zhao, Cheng Guo, Quanjun Yang

**Affiliations:** 1 Department of Pharmacy, Shanghai Sixth People’s Hospital Affiliated to Shanghai Jiao Tong University School of Medicine, Shanghai 200233, China; 2 Department of Clinical Pharmacy, Xin Hua Hospital Affiliated to Shanghai Jiao Tong University School of Medicine, Shanghai 200092, China

**Keywords:** breast cancer, nanomedicine, natural products, drug delivery, targeted therapy

## Abstract

**Background:** Breast cancer remains the most common malignant tumor among women worldwide. Although conventional treatments including surgery, chemotherapy, and radiotherapy are effective, they are confronted with challenges such as tumor heterogeneity, systemic toxicity, and recurrence driven by drug resistance. To overcome these limitations, natural products have emerged as promising therapeutic alternatives owing to their multi-target efficacy and favorable biocompatibility. However, their clinical translation is still hindered by poor chemical stability, low aqueous solubility, and inadequate bioavailability. Nanodelivery systems offer a transformative solution by enhancing bioavailability and enabling precision targeting through the enhanced permeation and retention effect, thereby widening the therapeutic window and minimizing off-target toxicity. **Purpose:** This review evaluates diverse nanoparticle-based delivery systems and their targeting mechanisms in natural product-based breast cancer therapy. By examining inherent advantages and translational challenges, this analysis provides critical insights into the clinical development and application of these nanoformulations. **Methods:** A systematic literature search was performed in PubMed, ScienceDirect, Springer, Taylor & Francis, and Web of Science to identify relevant studies on natural product-based nanoformulations for breast cancer therapy. **Results:** Natural products exert anti-breast cancer effects through mechanisms such as inducing apoptosis, arresting the cell cycle, inhibiting invasion and metastasis, suppressing angiogenesis, and regulating autophagy. To overcome clinical hurdles, three complementary targeting strategies have been developed: passive, active, and stimuli-responsive targeting. These advances are shifting nanomedicines toward active precision therapy, markedly improving the therapeutic index. With multiple formulations already approved or in clinical pipelines, this field is rapidly progressing from laboratory research to clinical implementation. **Conclusions:** Natural products possess potent anti-breast cancer effects, and the application of nanodelivery technology effectively overcomes their inherent limitations of poor stability and low bioavailability. Although preliminary findings are promising, large-scale, randomized controlled clinical trials are urgently needed to systematically evaluate their safety, efficacy, and practical potential for clinical translation in breast cancer management.

## 1. Introduction

**Figure 1:**
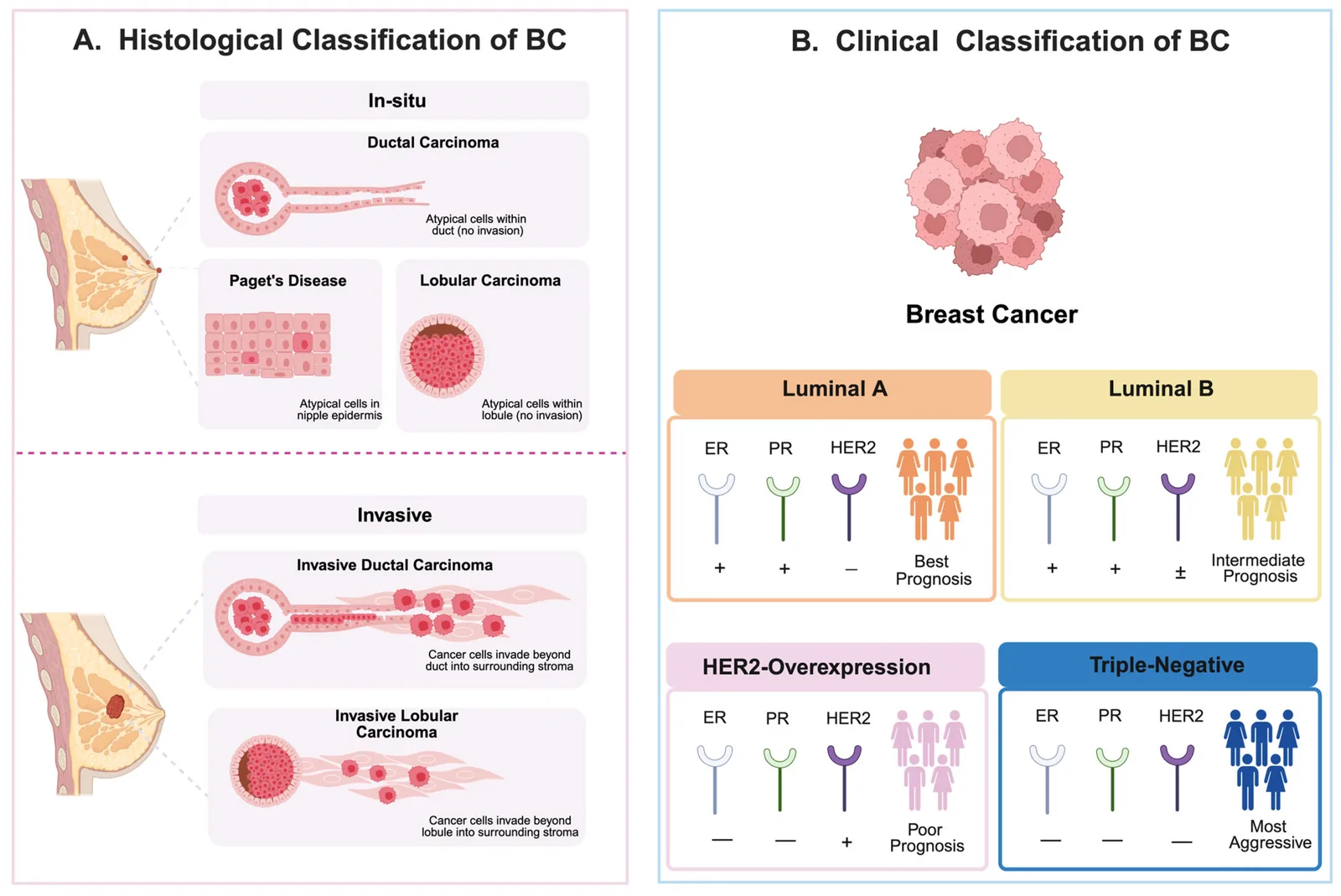
Histological and clinical classifications of BC. (**A**) Histologically, carcinomas are classified as in situ (lobular, ductal, and Paget’s disease) and invasive (predominantly invasive lobular and ductal carcinomas). (**B**) Clinically, tumors are categorized into four molecular subtypes based on ER, PR, and HER2 expression: Luminal A (best prognosis), Luminal B (intermediate prognosis), HER2-overexpression (poor prognosis), and triple-negative (worst prognosis).

Breast cancer (BC) is one of the most prevalent malignant tumors among women worldwide, accounting for approximately 30% of all female cancers and imposing a substantial burden on both patients and healthcare systems (Figure 1). The global incidence of BC has been steadily increasing in recent years, with projections indicating a 38% increase in new cases by 2050 [1,2]. Based on histological and molecular characteristics, BC can be classified into three main subtypes: approximately 60% of cases are positive for estrogen receptor (ER+) or progesterone receptor (PR+) which can be further subdivided into Luminal A and Luminal B subtypes based on proliferation indices (Ki-67) and HER2 status, 20% overexpress human epidermal growth factor receptor 2 (HER2^+^), and 10–20% are classified as triple-negative breast cancer (TNBC), characterized by the absence of ER, PR, and HER2 expression.

Among these subtypes, TNBC is notably more aggressive and associated with a higher risk of recurrence than other BC subtypes. Standard treatment modalities for BC include surgery, radiotherapy, and chemotherapy [3]. While surgery and radiotherapy are effective for localized tumors, their efficacy diminishes significantly with metastatic spread. Chemotherapy remains essential for systemic disease control, particularly for TNBC; however, its efficacy is constrained by drug resistance, relapse, and toxicity to healthy tissues [4]. Consequently, the development of novel therapeutic strategies featuring precise targeting, reduced off-target toxicity, and improved efficacy represents a critical priority in BC research.

Natural products have long been used to treat inflammatory, infectious, and malignant diseases [5]. Recent research has confirmed their efficacy against BC through mechanisms including induction of apoptosis, cell-cycle arrest, inhibition of angiogenesis and metastasis, and modulation of autophagy. Notably, approximately 60% of clinically approved anticancer drugs are derived from natural products, including paclitaxel (PTX), vinca alkaloids, etoposide, topotecan, and irinotecan. However, their clinical application in BC treatment is constrained by intrinsic physicochemical properties, including poor stability, low aqueous solubility, limited systemic absorption, low oral bioavailability, and non-specific tissue distribution [6].

To overcome the limitations of natural products and enhance their anticancer efficacy, researchers are dedicated to developing biodegradable and biocompatible nanosystems that can prolong circulation, improve stability, promote tumor-selective drug accumulation, and minimize off-target effects. This review provides a comprehensive overview of natural product-based nanotherapeutics for BC. First, it systematically summarizes the main mechanisms of action through which natural products exert their anti-BC effects. Next, based on the spatial relationship between natural products and nanocarriers, these nanoparticles are classified into encapsulated, derived, and functionalized types, with a comparison of their respective advantages and limitations. Subsequently, the three major targeting strategies of nano-based drug delivery systems, namely passive targeting, active targeting, and stimuli-responsive targeting, are described, along with a discussion of applicable scenarios for each strategy. Furthermore, given the molecular heterogeneity of BC, special attention is paid to personalized nanotherapeutic strategies for triple-negative, HER2-positive, and hormone receptor-positive subtypes. Finally, the current status of natural product-based nanomedicines that have entered clinical trials or received regulatory approval is reviewed. Translational bottlenecks at the manufacturing, biological, and regulatory levels are subsequently analyzed, followed by perspectives on future research directions.

## 2. Literature Search Strategy and Selection Criteria

A systematic literature search was conducted in PubMed, ScienceDirect, Springer, Taylor & Francis, and Web of Science. The search terms used included “natural products,” “phytochemicals,” “plant extracts,” “active ingredients,” “nanoformulations,” “nanoparticles,” “nano-drug delivery systems,” “nanocarriers,” “breast cancer,”and “breast carcinoma,” along with various combinations of these keywords. Study selection followed strict criteria. We included peer-reviewed original research articles in English that evaluated natural product-based nanomedicines specifically for BC. Non-original articles, studies on purely synthetic drugs or other cancers, and papers lacking sufficient nanoformulation characterization were excluded. After deduplication, two independent reviewers screened the titles and abstracts, and discrepancies were resolved by consensus with a third senior author.

## 3. The Anti-Breast Cancer Mechanisms of Natural Products

**Figure 2:**
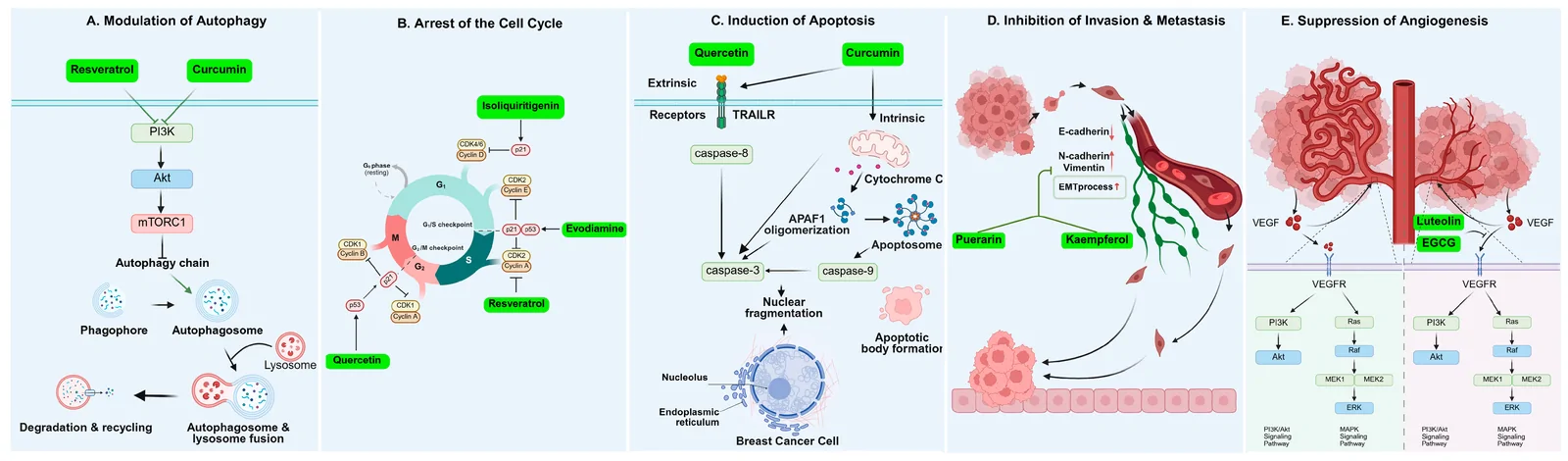
Pharmacological mechanisms of natural products against BC. (**A**) Autophagy modulation: Targeting the PI3K/Akt/mTOR signaling pathway and modulating Beclin1/Bcl-2 complexes. (**B**) Cell Cycle Arrest: Inducing G1/S or G2/M phase arrest by inhibiting cyclins, cyclin-dependent kinases (CDKs), and related regulatory proteins. (**C**) Apoptosis induction: Activating both extrinsic and intrinsic apoptotic pathways to ultimately trigger caspase-3 to induce cell death. (**D**) Metastasis inhibition: Suppressing tumor invasion and metastasis primarily via the blockade of the epithelial–mesenchymal transition. (**E**) Angiogenesis suppression: Preventing tumor neovascularization by blocking the VEGFR signaling pathway and its downstream targets.

Over the past several decades, natural products have emerged as an indispensable source for the development of novel anticancer drugs due to their significant antitumor activity and favorable safety profiles. This paper summarizes natural products that exert anti-breast cancer effects, as illustrated in Figure 2. Extensive evidence indicates that these natural products operate through five core pathways: induction of apoptosis, arrest of the cell cycle, inhibition of invasion and metastasis, suppression of angiogenesis, and modulation of autophagy (Figure 2).

BC progression often stems from the evasion of apoptosis. Natural products such as quercetin and curcumin (CUR) can activate either extrinsic or intrinsic apoptotic pathways, thereby restoring programmed cell death in cancer cells. Concurrently, compounds like apigenin and epigallocatechin gallate (EGCG) inhibit tumor proliferation by interfering with cyclin-dependent kinase (CDK) complexes, resulting in cell cycle arrest during the S and G2/M phases [7]. Furthermore, metastasis remains the leading cause of mortality in BC. Natural products, including flavonoids such as kaempferol and puerarin, have been shown to significantly reduce tumor invasion and migration by downregulating matrix metallopeptidase 2 (MMP-2) and -9 (MMP-9), as well as by inhibiting epithelial–mesenchymal transition (EMT) [8]. Tumor growth is also heavily reliant on pathological angiogenesis. EGCG and luteolin specifically target the vascular endothelial growth factor (VEGF) pathway to obstruct neovascularization, thereby depriving tumors of essential nutrients and oxygen [9,10]. Additionally, autophagy plays dual roles in BC, functioning as a tumor suppressor in early stages while promoting survival in advanced stages. CUR and resveratrol bidirectionally regulate autophagy by modulating the PI3K/AKT/mTOR pathway, thereby enhancing treatment sensitivity and mitigating stress-induced damage. Collectively, these mechanisms inhibit tumor growth, metastasis, and recurrence, thereby demonstrating the multi-target potential of natural products in the treatment of BC (Table 1).

## 4. Classification of Natural Product-Based NDDSs for Targeting BC

To overcome the aforementioned pharmacokinetic barriers of natural products, various nanosystems have been developed. In this review, natural product-based NDDSs are systematically categorized into three primary classes based on their functional roles and spatial distribution within the nanocarriers: encapsulated, derived, and functionalized nanoparticles. Specifically, natural products can serve as passive therapeutic payloads loaded into exogenous nanocarriers via encapsulation or surface adsorption, thereby shielding them from premature degradation. Alternatively, they can also transcend their role as simple cargo and act as the primary structural backbone that drives carrier self-assembly. Furthermore, when grafted onto the outer surface of these nanocarriers, they can improve the structural stability, biosafety, and photoresponsiveness of the nanoparticles, while also conferring synergistic therapeutic effects via their inherent pharmacological activities.

### 4.1. Natural Product-Encapsulated Nanoparticles

**Figure 3:**
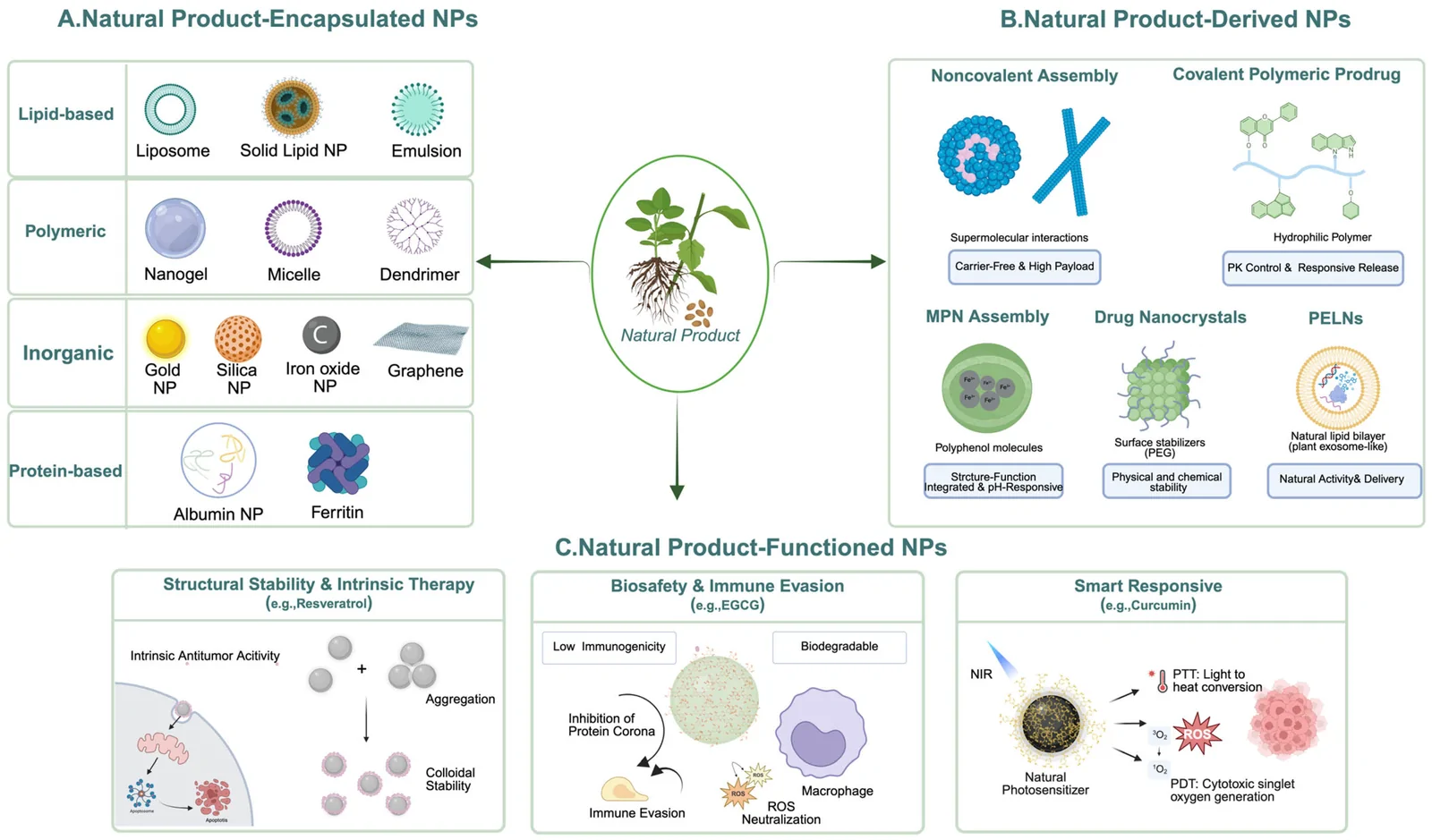
Classification and application strategies of natural product-based nanomedicine. (**A**) Natural product-encapsulated nanoparticles: Loading into conventional nanocarriers, including lipid-based, polymeric, inorganic, and protein-based systems. (**B**) Natural product-derived nanoparticles: Formulated as carrier-free noncovalent assemblies, covalent polymeric prodrugs, metal-polyphenol networks (MPNs), drug nanocrystals, and plant-derived exosome-like nanovesicles (PELNs). (**C**) Natural product-functionalized nanoparticles: Surface engineering to enhance structural stability (resveratrol), improve biosafety and immune evasion (EGCG), and enable smart, stimuli-responsive drug release (curcumin).

Within this structural category, natural products serve as passive therapeutic payloads that are loaded into and protected by conventional nanocarriers. Loading strategies include internal encapsulation and surface physical adsorption, which are driven by noncovalent forces including hydrophobic interactions, electrostatic adsorption, and hydrogen bonding. Common carriers include lipid-based, polymeric, inorganic, and protein-based nanocarriers (Figure 3A).

#### 4.1.1. The Lipid-Based Nanocarriers

Liposomes are composed of amphiphilic phospholipid bilayers, enabling the encapsulation of hydrophilic drugs or siRNAs in the inner aqueous core and hydrophobic drugs within the lipid bilayer. They exhibit excellent biocompatibility, can be readily surface-modified, and demonstrate prolonged circulation times in the bloodstream, making them an ideal nanocarrier for targeting breast cancer stem cells (BCSCs). As pioneering platforms in clinical translation, liposomes have achieved considerable success. For example, PEGylated liposomal doxorubicin, the first FDA-approved nanomedicine, exhibits a significantly improved cardiac safety profile in the treatment of metastatic BC compared with conventional formulations [29]. Nevertheless, the widespread clinical application of liposomes remains constrained by inherent limitations, including premature drug leakage, in vivo structural instability, and high costs associated with large-scale manufacturing.

Solid lipid nanoparticles (SLNs) are composed of a lipid matrix that remains solid at physiological temperatures. They exhibit excellent biodegradability, tissue compatibility, and low systemic toxicity. Compared with liposomes, SLNs demonstrate superior stability and encapsulation efficiency for hydrophobic drugs. However, their widespread application is limited by inherent drawbacks, including relatively low drug-loading capacities and the risk of premature drug leakage during storage. This leakage is primarily driven by the progressive crystallization and polymorphic transitions of the lipid matrix [30]. Although SLN-based formulations for BC have not yet entered clinical trials, preclinical studies highlighting the potent anti-BC efficacy of PTX-loaded SLNs underscore their considerable translational potential [31].

#### 4.1.2. The Polymer-Based Nanocarriers

Polymeric micelles are self-assembled nanoparticles formed from amphiphilic block copolymers. Compared with lipid-based systems, these platforms offer superior structural tunability and are characterized by an ultra-small size, which enables deeper interstitial penetration into the dense breast tumor microenvironment [32]. Their core–shell architecture effectively solubilizes highly hydrophobic natural products within the hydrophobic core, while the hydrophilic corona prevents rapid immune clearance and prolongs systemic circulation. However, their clinical translation is hindered by thermodynamic instability. Extreme dilution in the bloodstream can cause copolymer concentrations to fall below the critical micelle concentration, triggering structural dissociation and premature drug leakage. Despite these challenges, their translational potential remains significant. For example, Genexol^®^-PM demonstrated superior efficacy and reduced toxicity in Phase III BC trials, successfully achieving market approval in South Korea [33,34].

Unlike liposomes or conventional micelles, which often vary in size, dendrimers are highly uniform, branched polymers that offer precise control over drug loading [35]. This structural precision provides a major advantage in quality control and manufacturing. However, their clinical translation faces two main limitations: complex multi-step synthesis and surface toxicity. While neutral dendrimers are safe, positively charged variants can induce severe membrane disruption and subsequent cytotoxicity. Although incorporating a protective PEG coating significantly reduces this toxicity [36], it creates a new challenge. Specifically, the dense PEG layer can hinder deep penetration into dense breast tumors and impede rapid intracellular drug release. Despite these hurdles, dendrimers show strong translational potential in BC. For instance, PEGylated Poly-L-lysine (PLL) dendrimers delivering docetaxel (DTX) have successfully entered Phase II clinical trials for other solid tumors. Given that DTX is a cornerstone of BC therapy, the successful clinical validation of these dendrimers offers a highly promising blueprint for developing next-generation BC nanomedicines [37].

#### 4.1.3. The Inorganic Nanocarriers

Unlike soft organic carriers, metal nanoparticles offer superior stability and unique physical properties for stimuli-guided delivery [38] and direct reactive oxygen species (ROS)-mediated tumor killing [39]. However, their critical translational limitation is poor biodegradability, which poses a risk of long-term heavy metal accumulation and toxicity in off-target organs. Despite this clearance challenge, their clinical potential remains compelling. For instance, CYT-6091 (colloidal gold combined with TNF-α) successfully completed Phase I trials in solid tumors, including breast cancer, demonstrating that engineered metal platforms can effectively balance targeting efficacy and safety [40].

Compared with liposomes with limited capacity and non-porous metal NPs, mesoporous silica nanoparticles (MSNs) offer large surface areas and tunable pores for exceptionally high drug loading [41]. However, their clinical translation is significantly restricted by incomplete in vivo clearance and surface-exposed silanol groups, which can induce severe hemolysis, pulmonary toxicity, and nephrotoxicity. Although lagging behind organic carriers in clinical trials, MSNs demonstrate strong preclinical potential in BC. MSN formulations loaded with doxorubicin, epirubicin, or targeted nucleic acids exhibit significant antitumor efficacy in BC models [42], positioning them as highly promising candidates for future combination therapies.

#### 4.1.4. The Protein-Based Nanocarriers

Unlike synthetic polymers or inorganic materials that often trigger immune clearance, endogenous protein-based nanocarriers offer superior biocompatibility and unique receptor-mediated tumor targeting capabilities [43]. However, their clinical translation is frequently hindered by high manufacturing costs, batch-to-batch biological variability, and low mechanical stability, which poses a risk of premature drug leakage in the bloodstream. Despite these structural vulnerabilities, protein platforms exhibit an exceptional clinical track record in BC therapy. Notably, Abraxane^®^ achieved FDA approval in 2005 for metastatic breast cancer by eliminating toxic solvents and enhancing tumor accumulation [44].

#### 4.1.5. Critical Comparison and Translational Potential of Nanoplatforms

Despite the robust preclinical efficacy demonstrated by various natural product-loaded nanocarriers, their clinical translation is consistently hindered by manufacturing hurdles and complex biological barriers. Among these, organic vehicles such as liposomes and protein-based platforms lead in clinical translation owing to their excellent biocompatibility and tumor-homing abilities, but they are limited by scalability issues and storage instability [45]. Conversely, SLNs and polymeric micelles offer cost-effective scalability for hydrophobic payloads, although micelles require cross-linking or stimuli-responsive designs to prevent dilution-induced premature dissociation [46]. Meanwhile, rigid platforms encompassing dendrimers, MSNs, and metal nanoparticles excel in precise drug loading and multifunctional theranostic applications. Nevertheless, their widespread translation is bottlenecked by the synthetic complexity and amine toxicity inherent to dendrimers [47], as well as the non-biodegradability and chronic tissue accumulation characteristic of inorganic carriers [48]. To systematically evaluate and summarize these structural, functional, and clinical dimensions, a comprehensive critical comparison of the relative advantages, limitations, and translational potential of these nanocarriers in breast cancer therapy is outlined in Table 2.

### 4.2. Natural Product-Derived Nanoparticles

The construction of natural product-derived nanoparticles can be classified into five categories: noncovalent pure drug assemblies, covalent polymeric prodrugs, metal ion coordination-driven assemblies, drug nanocrystals, and plant-derived exosome-like nanovesicles (PELNs) (Figure 3B). In the noncovalent pure drug assembly approach, natural products rely solely on intermolecular forces to spontaneously form nanoparticles [49]. This completely carrier-free design achieves exceptionally high drug loading while thoroughly eliminating the toxicity risks associated with exogenous carriers [50]. Guo and colleagues highlighted that specific flavonoid or alkaloid natural products can form nanofibers or nanospheres through supramolecular interactions [51]. In BC applications, these spontaneously assembled structures serve not merely as drug delivery vehicles but also inherently possess biological activities capable of disrupting cancer cell signaling pathways. The covalent polymeric prodrug strategy involves chemically grafting natural products onto hydrophilic polymer backbones [52]. This architecture significantly enhances systemic circulation stability and enables precise microenvironment-responsive drug release. For example, Ren et al. covalently grafted docetaxel and the HIF-1α inhibitor YC-1 onto hydrophilic polymers via a cathepsin B-sensitive peptide linker. The co-assembly of these prodrugs enabled specific dual-drug release within the TNBC microenvironment, synergistically enhancing neoadjuvant chemotherapy while significantly reducing systemic toxicity [53].

Metal ion coordination-driven assembly relies on interactions between polyphenolic catechol groups and transition metal ions to construct metal-polyphenol network (MPN) nanoparticles in a single step. Polyphenols serve as both structural scaffolds and therapeutic agents, exhibiting pH-responsive dissociation within the acidic tumor microenvironment [54]. For example, Fan et al. co-loaded metformin and glucose oxidase into EGCG/Fe^3+^ nanoparticles, achieving synergistic chemodynamic and metabolic therapy [55]. The drug nanocrystal strategy directly formulates natural products into nanoscale crystals stabilized by minimal amounts of surfactants or polymers (such as PEG) to prevent aggregation. Notably, Zhao et al. developed folic acid/PEG dual-modified paclitaxel nanocrystals (PTX-PEG-FA), which significantly prolonged the blood circulation time of paclitaxel, enhanced intratumoral drug accumulation, and exhibited superior tumor growth inhibition compared with both Taxol and unmodified nanocrystals [56].

Unlike artificial assemblies, PELNs are naturally secreted membranous vesicles rich in proteins, lipids, RNAs, and secondary metabolites. Their inherent polyphenols, miRNAs, and biolipids exert direct anti-proliferative, pro-apoptotic, and drug-resistance-reversing effects, while also modulating the tumor microenvironment through immune cells [57]. Additionally, their lipid bilayers facilitate exogenous drug loading and transmembrane delivery. For example, Chen et al. demonstrated that natural nanovesicles isolated from tea flowers inhibited BC metastasis via a dual mechanism involving ROS generation and gut microbiota modulation [58].

### 4.3. Natural Product-Functionalized Nanoparticles

The core of natural product-functionalized nanoparticles is that natural products not only significantly enhance the structural stability, biosafety, and photoresponsiveness of the nanoparticles but also endow them with the synergistic effects of their intrinsic pharmacological activities (Figure 3C). First, natural product modification layers play a critical role in the structural stability of nanoparticles. For instance, a resveratrol-based natural product modification layer can effectively overcome the physical vulnerability of metallic nanoparticles to aggregation, providing them with excellent colloidal dispersion stability. More notably, natural products serving as modification layers often possess potent intrinsic pharmacological activities. Resveratrol not only endows the nanosystem with mitochondrial targeting capabilities, but also exhibits significant dose-dependent antitumor activity against BC cells [59].

Second, natural product modification layers play a key role in enhancing the in vivo biosafety of nanosystems. Upon entering the systemic circulation, nanoparticles are rapidly coated by serum proteins to form a “protein corona,” which triggers clearance by the mononuclear phagocyte system (MPS) and ultimately alters their in vivo fate and biodistribution [60]. Polyphenolic natural products such as EGCG and tannic acid inhibit nonspecific protein adsorption and hinder protein corona formation through their hydrophilic surfaces, thereby significantly reducing MPS recognition and clearance rates [61]. Compared with conventional PEGylation strategies, natural products offer the dual advantages of biodegradability and low immunogenicity. Moreover, their inherent antioxidant activity can neutralize oxidative stress byproducts in the circulation, mitigating collateral damage to healthy tissues [62].

In addition, natural products can endow nanocarriers with unique smart responsiveness. For example, in photoresponsive design, Ashkbar et al. developed a composite system that integrates curcumin as a photosensitizer with magnetic nanoparticles [63]. In this system, curcumin not only acts as an active antitumor molecule but also as a crucial photosensitive functional module, enabling in vivo treatment of breast cancer through dual-modal photodynamic and photothermal therapies. Furthermore, broadly defined naturally derived materials exhibit tremendous potential in nanocarrier surface engineering. For instance, the use of natural shellac for the surface modification of carbon nanotubes can significantly improve their aqueous dispersibility and effectively shield the intrinsic toxic sites of the inorganic carriers [64]. Moreover, the use of natural DNA molecules to precisely control the surface co-precipitation assembly of amorphous calcium phosphate (ACP) can balance delivery efficiency and cytocompatibility by optimizing the size and surface density of the nanospheres [65].

Overall, this innovative strategy of using naturally derived materials to modify nanoparticle surfaces and mitigate toxicity offers a groundbreaking perspective on the clinical translation of nanomedicine systems. On the one hand, this strategy breaks the limitation of traditional carriers acting merely as inert transport vehicles. The modification layer simultaneously serves multiple protective and therapeutic functions, fundamentally simplifying the complexity of nanoformulations and greatly reducing the difficulty of quality control during large-scale production [66]. On the other hand, the introduction of natural molecules with superior biocompatibility is expected to gradually replace conventional synthetic surfactants such as polyethylene glycol (PEG). This not only ingeniously circumvents the risks of immunogenicity and allergic reactions potentially triggered by synthetic excipients but also ensures that the materials are safely metabolized into harmless small molecules in vivo, thereby removing major safety hurdles for regulatory approval and clinical translation.

## 5. The Mechanisms of Tumor Targeting in Nano-Based Drug Delivery Systems

**Figure 4:**
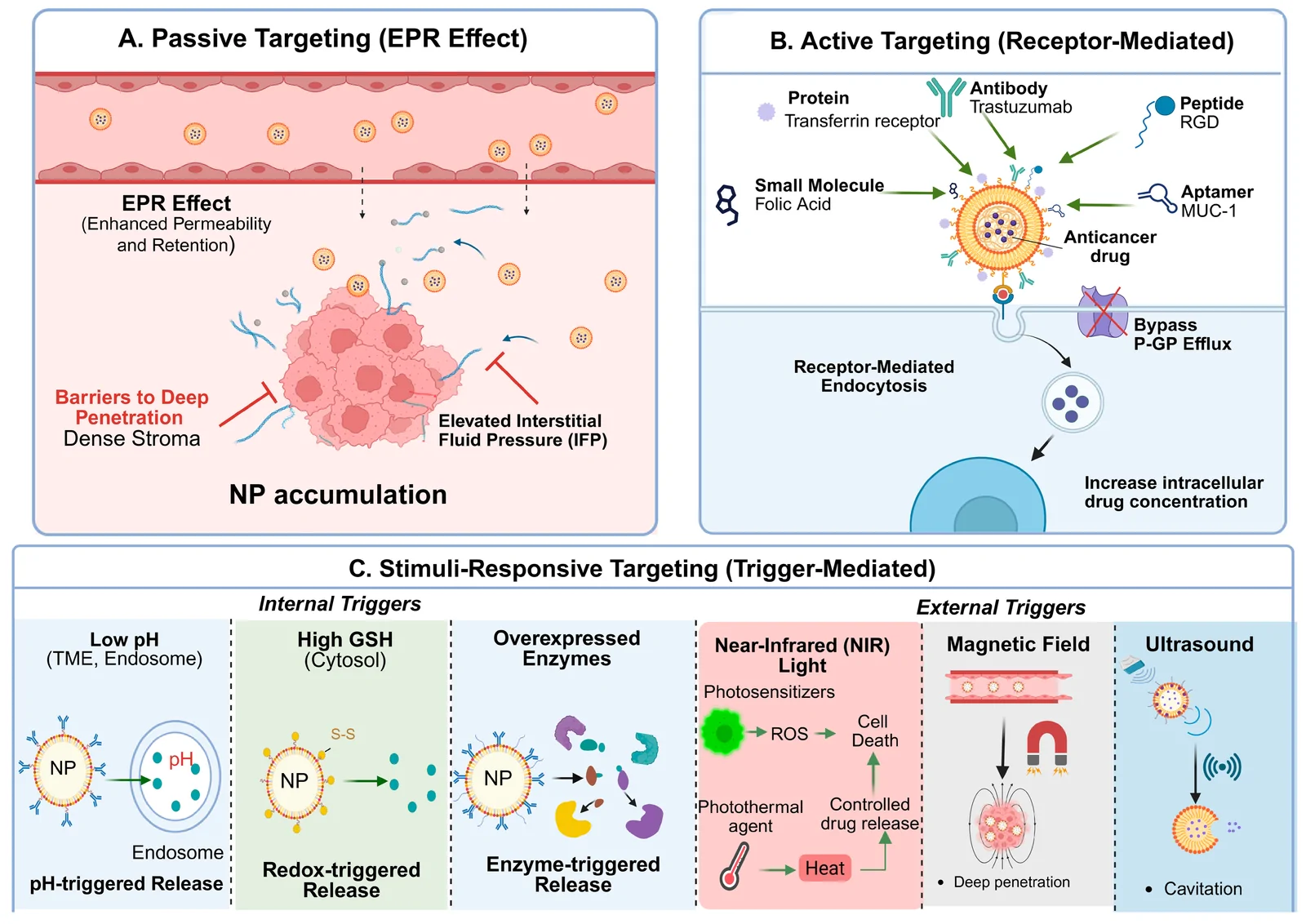
Mechanisms of tumor targeting in nano-based drug delivery systems. (**A**) Passive targeting. Nanoparticles accumulate in the tumor site via the enhanced permeability and retention (EPR) effect through leaky vasculature. (**B**) Active targeting. Nanocarriers functionalized with specific surface ligands facilitate receptor-mediated endocytosis. (**C**) Stimuli-Responsive Targeting. Smart nanocarriers undergo physicochemical transformations to release payloads in response to specific triggers, overcoming physical and biological barriers.

The preceding section classified NDDSs based on the spatial configuration and structural roles of natural products. To elucidate how these systems achieve therapeutic selectivity, the focus now shifts from a materials-centric perspective to a mechanism-driven approach. As illustrated in Figure 4, nanodelivery systems achieve precise localization through three complementary strategies: passive targeting driven by the enhanced permeability and retention (EPR) effect, active targeting mediated by ligand-receptor recognition, and stimuli-responsive targeting triggered by the tumor microenvironment.

### 5.1. Passive Targeted NDDS

Traditionally, passive targeting relies on the EPR effect, which enables nanoparticles with hydrodynamic diameters of 100 to 400 nm to exploit abnormal angiogenesis, leaky vasculature, and compromised lymphatic drainage to achieve selective tumor accumulation. Table 3 summarizes various natural product-based NDDS that employ passive targeting mechanisms for breast cancer. However, as currently debated in the field, the clinical relevance and broad applicability of the EPR phenomenon in human oncology remain highly controversial. Although this effect is pronounced in murine models, human EPR is severely restricted by intertwined biological and physical barriers. Biologically, human tumor vasculature exhibits extreme heterogeneity with erratic permeability, and the mononuclear phagocyte system (MPS) prematurely clears most nanoparticles from circulation [67]. Physically, the EPR effect only drives vascular leakage rather than deep penetration. Extravasated nanoparticles immediately confront dense desmoplastic stroma and elevated interstitial fluid pressure (IFP). This intense mechanical resistance leads to severe perivascular trapping, preventing drugs from reaching deep-seated cancer cells. A meta-analysis of nanoparticle delivery efficiency reported that only a median of 0.7% of administered nanoparticles reached solid tumors. Although this specific statistical conclusion provoked intense academic debate regarding its methodology, it undeniably prompted a critical reevaluation of purely passive targeting strategies and highlighted the fundamental inadequacy of relying solely on the unpredictable natural EPR effect [68].

To overcome the profound heterogeneity of the native vascular barrier, cutting-edge delivery strategies have pivoted toward artificially enhancing vascular permeability. For instance, the super-enhanced permeability and retention (SUPR) effect mediated by photoimmunotherapy utilizes specific photosensitizers combined with near-infrared irradiation to achieve rapid and highly localized vascular opening [69]. This mechanism not only increases the tumor accumulation of various nanomaterials by up to 24-fold but also remains completely independent of their physicochemical properties. Furthermore, researchers have begun shifting toward active targeting and stimuli-responsive strategies.

**Table 3:** Some of the natural product-based NDDS through passive targeting mechanisms proposed for BC.

Nano-Carrier	Natural Product (Co-Loaded)	Size (nm)	Drug Loading (%)	Experimental Design	Key Findings	Ref
Liposome	PTX (Anti-CD47)	~120	9.0	In vitro (MDA-MB-231 and RAW264.7 cells) In vivo (MDA-MB-231 tumor-bearing BALB/c mice)	Enhances phagocytosis of tumor cells and activates the systemic T-cell immunological response	[70]
Lipid nanocapsule	PTX (Salinomycin)	89 ± 3	PTX: 0.202 ± 0.004 SAL: 4.12 ± 0.08	In vitro (MCF-7 cells)	Induces apoptosis in CSCs and reduces the growth of tumor mammospheres	[71]
Polymer–lipid hybrid nanoparticles	PTX (Verteporfin, combretastatin)	100~150	NA	In vitro (MDA-MB-231 cells) In vivo (Transgenic zebrafish model, Athymic mice, patient-derived xenograft)	Downregulates YAP target genes, decreases the activity of HIF-1α and the gene expression of VEGF, and inhibits bulk cancer cells, CSCs, and angiogenesis	[72]
Calcium phosphate composite lipid nanosystem	PTX (miR-124)	187.4 ± 5.6	NA	In vitro (MDA-MB-231 cells) In vivo (MDA-MB-231 tumor-bearing BALB/c nude mice)	Promotes apoptosis and inhibits migration and invasion	[73]
Nucleobase-crosslinked poly(2-oxazoline) nanoparticles	PTX	39.7	38.3	In vitro (E0771 and 4T1 cells) In vivo (Xenograft: BALB/c and C57BL/6 mice, 4T1 and E0771 cells)	Prolongs blood circulation and enhances tumor accumulation of PTX	[74]
Branched polymeric prodrug nanoparticles	PTX (Cyanine5.5 and gadolinium chelates)	62.6 ± 1.1	NA	In vitro (4T1 cells) In vivo (4T1tumor-bearing mice)	Impairs the function of microtubules	[75]
HCQ- and Fmoc-modified HES nanogels	PTX	182.5 ± 4.6	NA	In vitro (4T1 cells) In vivo (Xenograft: BALB/c mice, 4T1 cells)	Prevents tumor cells from metastasizing to the lungs, inhibits excessive autophagy, and enhances cell adhesion	[76]
NIR-nanogels	PTX	110 ± 35	NA	In vitro (4T1 cells) In vivo (Xenograft: BALB/c mice, 4T1 cells)	Inhibits tumor growth and reduces tumor metastasis	[77]
Polymeric micelles	PTX (ATN peptide)	42.5 ± 1.6	16.7	In vitro (4T1 cells) In vivo (4T1 tumor-bearing mice)	Inhibits primary tumor cells and mitigates lung metastases	[78]
High-capacity poly(2-oxazoline) (POx)-based polymeric micelles	PTX (PLX3397)	57	PTX: 21.2 PLX3397: 22.5	In vitro (4T1, T11-Apobec, and T12 cells) In vivo (4T1 tumor bearing BABL/c mice)	Inhibits the growth and metastasis of primary tumors	[79]
Outer membrane vesicles	PTX (siRNA)	NA	NA	In vitro (4T1 cells) In vivo (Xenograft: BALB/c mice, 4T1 cells)	Regulates the triple-negative breast cancer metabolic microenvironment and suppresses tumor growth	[80]
Albumin nanoparticle	PTX (IPI-549)	143.5 ± 2.0	8.1 ± 0.04	In vitro (4T1 cells) In vivo (MMTV-PyMT transgenic mice with spontaneous breast cancer)	Reshapes the immune microenvironment to inhibit tumor growth and eliminates lung metastasis	[81]
PLGA/TPGS nanoparticles	DTX (SAL)	73.83	SAL: 4.08 DTX: 4.12	In vitro (MCF-7 cells, MCF-7-MS) In vivo (Xenograft: BALB/c nude mice, MCF-7 cells)	Prolongs circulation time and inhibits breast cancer cells and CSCs	[82]
CM-camouflaged and USIO nanoparticle-loaded polyethylenimine nanogels	DTX (siCD47)	203	11.20	In vitro (4T1, L929, DCs, and RAW264.7 cells) In vivo (Xenograft: BALB/c mice, 4T1 cells)	Restores phagocytic activity and inhibits the growth of primary breast cancer and lung metastases	[83]
Liposome	DTX (Curcumin)	208.53 ± 6.82	NA	In vitro (MCF-7 cells) In vivo (Xenograft: nude mice, MCF-7 cells)	Enhances anti-tumor effects and reverses the drug resistance of DTX	[84]
Core-shell-structured liposome	Curcumin (PTX)	514	NA	In vitro (MDA-MB-231 cells)	Improves cellular internalization and enhances the anticancer effect	[85]
Chondroitin sulfate-based micellar nanogel	Curcumin	63.08 ± 13.02	6.55 ± 2.88	In vitro (MCF-7 cells)	Inhibits BC cell proliferation, blocks the cell cycle, and induces apoptosis	[86]
Silk fibroin nanospheres	Apigenin	163.35	6.20 ± 0.25	In vitro (4T1 and MDA-MB-231 cells)	Prolongs the half-life of apigenin and improves its bioavailability	[87]
Polymer–lipid hybrid nanoparticles	Apigenin	125.73 ± 5.57	7.56 ± 0.27	In vitro (MCF-7 and MDA-MB-231 cells)	Prolongs the time of drug release and decreases cell viability	[88]
mPEG-PLGA	Baicalein	195.5 ± 23	11	In vitro (4T1, NIH3T3 cells) In vivo (Xenograft: BALB/c mice,4T1 and NIH3T3 cells)	Inhibits the TGF-β signaling pathway and activates the tumor immune microenvironment	[89]
Chitosan/silver nanocomposite	Kaempferol	7~10	NA	In vitro (MDA-MB-231 cells)	Induces apoptosis in MDA-MB-231 cells	[90]
Chitosan-functionalized copper oxide nanoparticles	Quercetin	50 ± 3	NA	In vitro (MCF-7, CaCo-2 and HepG-2 cells) In vivo (DMBA-induced rats)	Induces apoptosis, prevents cell cycle progression, and inhibits the expression of PCNA	[91]
Nanoparticles integrated with galactan coating and mesoporous polydopamine, equipped with 2,2,6,6-Tempo	Quercetin (Dasatinib)	147.20 ± 22.17	Dasatinib: 18.08 ± 0.18 Quercetin: 45.71 ± 1.50	In vitro (4T1, A549 cells) In vivo (4T1 tumor-bearing mice)	Removes senescent tumor cells induced by chemotherapy, thereby preventing the growth and metastasis of breast cancer	[92]
Fe^3+^-mediated nanoassemblies	Quercetin (Mitoxantrone)	134 ± 10	Mitoxantrone: 55.9 Quercetin: 19.0	In vitro (4T1 cells) In vivo (4T1 tumor-bearing BALB/c mice)	Regresses the growth of the primary tumor and can also effectively inhibit distal breast tumor recurrence	[93]
NCG nanoparticles	Naringenin	142.89 ± 0.94	40.65	In vitro (WRL-68, MCF-7, Caco-2, HUVEC, E0771, and HBL100 cells) In vivo (Zebrafish breast cancer xenograft model, ER-positive E0771 tumor-bearing C57BL/6 female mice)	Increases the expression of EST and decreases estradiol levels in the liver, blood, and tumor tissues	[94]
Nitroimidazole-modified hyaluronic acid–oxalate	All-trans retinoic acid (ATRA)	150	CPT: ~7.3 ATRA: ~6.7	In vitro (MCF-7, HepG2 and PANC-1cells) In vivo (xenograft: BALB/c nude mice and Wistar rats, MCF-7 cells;NOD/SCID mice, HCC70 and SUM159PT tumorsphere cells mixture; BALB/c mice, 4T1-Luc adherent and tumorsphere cells)	Inhibits tumor growth and reduces stemness-related recurrence and metastasis	[95]
Nanostructured Lipid Carriers	Resveratrol	104.47	NA	In vitro (MDA-MB-231 cells) In vivo (Female Sprague–Dawley rats)	Higher cytotoxicity and cellular uptake efficiency, and inhibits cell migration	[96]
mPEG-DSPE-Vitamin E TPGS-Lipid nanocapsules	Thymoquinone (DTX)	99.06 ± 2.23	TQ: 2.61 ± 0.04 DTX: 1.19 ± 0.05	In vitro (MCF-7 and MDA-MB-231 cells) In vivo (Ehrlich ascites carcinoma-bearing mice)	Increases sensitivity to DTX, improves anti-metastasis effect, and enhances anti-tumor effect	[97]
D-α-tocopherol polyethylene glycol succinate (TPGS) nanoparticles	Berberine, Lonidamine, Gambogic acid	NA	BBR: 25.97 ± 1.34 LND: 9.01 ± 0.21 GA: 34.86 ± 3.08	In vitro (MCF-10A, MDA-MB-231 cells) In vivo (xenograft: BALB/c nude mice, MDA-MB-231 cells)	Increases ROS production and reduces MMP production, thereby enhancing the inhibitory effect on tumor proliferation and migration.	[98]
PLGA-based nanoparticles	Piperlongumine	251 ± 0.6	~9.5	In vitro (MDA-MB-231 cells)	Inhibits the self-renewal, chemoresistance, and epithelial-mesenchymal transition of CSCs by downregulating STAT3	[99]
CS-coated LPHNPs	Thymoquinone	179.63 ± 4.77	NA	In vitro (MDA-MB-231 and MCF-7 cells)	Enhances oral bioavailability as well as therapeutic efficacy	[100]
CS-coated PLGA nanoparticles	Thymoquinone	125	2.66	In vitro (MDA-MB-231 and SUM-149 cells)	Enhances anti-cancer effects	[101]
Aragonite Calcium Carbonate Nanoparticles	Thymoquinone (Doxorubicin)	NA	NA	In vitro (MDA-MB-231 cells)	Destroys breast cancer stem cells, reduces the activity of ALDH and the expression of CD44 and CD24	[102]
Pluronic F127 Micelles	Citral	26.51 ± 0.32	9.95 ± 0.01	In vitro (MCF-7, MDA-MB-231, MDA-MB-468 and HCC-1806 cells)	Inhibits the proliferation of breast cancer cells and effectively blocks the stem cell properties of cancer stem cells	[103]

### 5.2. Active Targeted NDDS

Active targeted NDDSs utilize surface ligands (such as antibodies, peptides, aptamers, proteins, and small molecules) to drive receptor-mediated endocytosis, effectively overcoming the limitations of passive accumulation. Crucially, this endocytic pathway bypasses P-glycoprotein (P-gp)-mediated multidrug efflux, thereby maximizing intracellular drug concentrations while minimizing off-target toxicity [104]. Table 4 summarizes various natural product-based NDDS that employ active targeting mechanisms for BC.

However, active targeting alone cannot overcome macroscopic physical barriers. Before surface ligands can exert their functions, nanoparticles must still navigate through dense tumor stroma and overcome elevated IFP. More problematically, active targeting strategies inherently trigger a severe binding-site barrier effect. Specifically, nanocarriers equipped with high-affinity ligands bind excessively to peripheral cancer cells surrounding the vasculature, thereby severely impeding their diffusion and penetration into deeper tumor regions. Consequently, although active targeting significantly enhances drug uptake at the cellular level, it fundamentally requires integration with stimuli-responsive mechanisms to achieve comprehensive spatial eradication of tumors.

#### 5.2.1. Peptide-Modified NDDS

##### RGD Peptide

The Arg-Gly-Asp (RGD) peptide and their derivatives serve as classic ligands for constructing targeted nanodelivery systems. By specifically recognizing integrins (such as αvβ3) that are highly expressed on tumor and vascular endothelial cells, they enable active targeted drug delivery, benefiting from their excellent biocompatibility. iRGD peptides, optimized from the basic RGD structure, interact simultaneously with integrins and Neuropilin-1 (NRP-1), thereby significantly enhancing tumor penetration and cellular uptake. This property makes them particularly suitable for scenarios that require disruption of matrix barriers. This method can be utilized for the precise delivery of drugs such as PTX [105] and can be integrated with photothermal therapy (PTT), chemotherapy, ferroptosis, and other strategies to achieve a synergistic treatment effect [106]. However, the complexity of multiple components poses the most stringent regulatory challenges.

Furthermore, cyclized cRGD markedly improves affinity and stability, making it a common choice for constructing multifunctional nanoplatforms that integrate chemotherapy, photothermal therapy, and tumor imaging capabilities [107,108]. It offers advantages in manufacturing reproducibility and clinical translation potential, but lacks active penetration capability. Additionally, combining the reverse RGD sequence (dGR) with cell-penetrating peptides (e.g., R9) enables the design of bifunctional peptides that exhibit high-efficiency targeting and deep penetration. Ma et al. engineered an R9dGR-peptide-modified active-targeting nanosystem co-loaded with gallic acid (GA) and PTX, significantly enhancing both tumor targeting and tissue penetration [109]. However, the cationic nature of R9 may induce nonspecific adsorption of serum proteins and rapid clearance, resulting in the lowest translational maturity. These RGD-based targeting strategies effectively enhance the tumor accumulation and therapeutic efficacy of nanomedicines, providing essential tools for precision synergistic cancer therapy.

##### CREKA Peptide

CREKA (Cys-Arg-Glu-Lys-Ala) is a short peptide that can target and selectively bind to microthrombi rich in fibrin in the tumor microenvironment, especially those formed after photodynamic therapy (PDT) induces vascular dysfunction. For example, Yang’s team developed an NDDS that integrates PDT-induced coagulation with CREKA peptide targeting. RGD-modified pyropheophorbide-a (Ppa) micelle (PPRM) facilitates localized thrombosis at tumor vasculature sites. Concurrently, circulating CREKA-modified NNDS specifically recognize and accumulate at these thrombus sites, significantly enhancing chemotherapy efficacy in BC [110]. The target of CREKA is abundantly present in tumor vasculature and stroma and can be further amplified through interventions such as photodynamic therapy (PDT). However, unlike iRGD, which relies on NRP-1-mediated natural transcytosis, the CREKA strategy depends on an artificially induced coagulation-targeting cascade that requires more stringent temporal control. Moreover, the long-term safety of PDT-induced thrombosis in patients with coagulation abnormalities remains to be assessed. Therefore, clinical translation of this strategy must prioritize addressing the two critical issues of temporal controllability and coagulation safety.

##### SILY Peptide

SILY (RRANAALKAGELYKSILYGC) is a short peptide that exhibits a high binding affinity and specificity for binding to type I collagen. In the context of BC, this extracellular matrix protein is overexpressed in the tumor stroma, creating a dense and accessible target for therapeutic interventions. SILY-modified hybrid NPs significantly enhance local retention and selectivity for tumor cells by targeting the overexpressed type I collagen in the breast tumor stroma. This mechanism facilitates potential intravascular delivery and localized therapy for BC [111]. Unlike RGD-mediated systemic intravenous delivery, the SILY platform is currently designed primarily for intraductal local administration, where its targeting signal serves “retention”. Consequently, it is unsuitable for systemic treatment of metastatic breast cancer but is better suited for local precision intervention in early-stage focal lesions, such as ductal carcinoma in situ (DCIS).

##### LHRH Peptide

Luteinizing hormone-releasing hormone (LHRH) receptors are predominantly expressed in pituitary gonadotroph cells, where they play a crucial role in regulating sex hormone secretion. These receptors are abnormally overexpressed in various tumors, including TNBC, with expression levels reaching several to dozens of times higher than those observed in normal tissues. In response to this phenomenon, researchers have developed an LHRH-targeted redox-responsive nanosystem. This innovative system is designed to specifically release PTX within the TNBC microenvironment, thereby significantly enhancing drug uptake and accumulation within tumor cells [112]. As an endogenous peptide hormone, LHRH has the advantage of more extensive systemic toxicology data compared with exogenous targeting peptides, which is favorable for clinical translation. However, its receptor plays essential physiological roles in the pituitary–gonadal axis, and systemic administration may disrupt normal endocrine regulation, potentially leading to sex hormone imbalances.

##### PD-L1 Binding Peptide

Programmed death-ligand 1 (PD-L1) is an immune checkpoint protein that plays a crucial role in regulating immune activity during normal inflammation. Its expression levels increase in BC cells following chemotherapy or radiotherapy. Upon binding to programmed cell death 1 (PD-1) on T cells, PD-L1 directly induces T cell exhaustion and suppresses antitumor immunity within the tumor microenvironment [113]. Modifying human serum albumin-CUR NPs with PD-L1 binding peptides achieves dual effects: it competitively blocks the PD-1/PD-L1 pathway to reverse T-cell exhaustion, while also releasing CUR to induce immunogenic cell death in tumor cells. This process promotes antigen release and systemic immune activation [114]. However, PD-L1 expression exhibits marked heterogeneity both intratumorally and intertumorally, and is dynamically modulated by chemotherapy and radiotherapy. As a result, targeting efficiency may fluctuate with the evolving therapeutic window, underscoring the need to prioritize in vivo persistence in clinical translation.

##### LyP-1 Peptide

LyP-1 (CGNKRTRGC) is a peptide that selectively targets the p32 protein, which is highly expressed on the surface of tumor cells and within mitochondria. Furthermore, LyP-1 exhibits a strong affinity for tumor blood vessels and lymphatic vessels. The LyP-1-modified nanocarrier effectively penetrates deep into tumor tissue via p32-mediated endocytosis, thereby enhancing delivery precision. Zhang et al. developed LyP-1-functionalized regenerated silk fibroin NPs encapsulating quercetin. This system results in mitochondrial damage and significantly inhibits the proliferation of BC cells as well as lung metastasis [115]. The core advantage of LyP-1 lies in its ability to simultaneously recognize tumor cells, tumor-associated macrophages, tumor vasculature, and lymphatic vessels, thereby conferring unique value in suppressing metastasis.

#### 5.2.2. Protein-Modified NDDS

Transferrin (Tf) receptors are transmembrane proteins that facilitate cellular iron uptake and are widely expressed on the surfaces of various cells. Notably, their expression in tumor cells can be 4 to 5 times higher than in normal cells. Leveraging this property, Tf-conjugated nanocarriers can bind to these receptors, triggering endocytosis and facilitating the delivery of drug-loaded complexes into the cells. For example, Xu et al. constructed Tf/pH-sensitive lipid bilayer-co-modified metal–organic framework (MOF) nanocarriers loaded with piperonamide (PL). This innovative approach enhances intracellular iron accumulation through Tf-mediated endocytosis, releases PL in the acidic tumor microenvironment to induce ferroptosis and promote reactive oxygen species (ROS) generation, and synergistically triggers ferroptosis and apoptosis under thermal stress [116]. While full-length proteins offer superior receptor affinity compared to short peptides, their large molecular weight may impede tissue penetration and increase immunogenicity. Beyond these targeting limitations, the therapeutic strategy itself presents clinical challenges. Although dual induction of ferroptosis and pyroptosis confers potent antitumor efficacy, it may elicit systemic inflammatory responses with a narrow therapeutic window. Moreover, the long-term in vivo degradation behavior and metal ion release kinetics of MOF carriers remain to be systematically evaluated.

#### 5.2.3. Antibody-Modified NDDS

Antibodies exhibit highly specific targeting capabilities, allowing for precise binding to cell surface antigens. This specificity significantly enhances drug accumulation at the lesion site and facilitates efficient intracellular delivery via receptor-mediated endocytosis. Moreover, therapeutic antibodies possess dual functions: they can block signaling pathways and recruit immune effector cells. These actions work synergistically to modulate the tumor microenvironment, thereby enhancing overall therapeutic efficacy [117]. However, their massive molecular weight severely restricts deep tissue penetration within the dense BC stroma, while the significant steric hindrance introduced by antibody conjugation can destabilize nanocarriers and accelerate clearance by the mononuclear phagocyte system (MPS). Consequently, their clinical translation urgently demands sophisticated carrier engineering to reconcile the inherent trade-off between exceptionally high receptor affinity and the physical permeability of solid tumors.

##### tmTNF-α

Given that transmembrane tumor necrosis factor-alpha (tmTNF-α) is highly expressed in approximately 84% of TNBC patients, Liu et al. developed a monoclonal antibody–PTX conjugated NP targeting tmTNF-α. This system significantly inhibited tumor growth in MDA-MB-231 tumor-bearing mouse models by inducing apoptosis, synergistically regulating NF-κB and PI3K/Akt pathways, and suppressing EMT, thereby effectively blocking tumor progression and metastasis [118]. Notably, tmTNF-α is expressed at a substantially higher rate than PD-L1 and HER2 in TNBC, making it an attractive target for this subtype, which otherwise lacks well-defined molecular targets. Compared with peptides such as RGD and LHRH, monoclonal antibodies are two orders of magnitude larger, conferring higher affinity and specificity but also limiting tumor penetration and increasing immunogenicity risk. Moreover, mAb production requires CHO cell expression and rigorous purification, leading to manufacturing costs far exceeding those of chemically synthesized peptide ligands and thus hindering large-scale clinical translation.

##### Trastuzumab

In studies of trastuzumab-modified active targeting systems, researchers developed trastuzumab-modified dual-stimulus-responsive mesoporous silica NPs. These carriers utilize MSNs as the core and co-load PTX and gemcitabine (Gem). The outer layer is composed of pH/heat dual-sensitive liposomes, which are covalently conjugated with trastuzumab. This carrier specifically targets and internalizes into HER2-positive SKBR3 cells. Under the acidic conditions of lysosomes and thermal stimulation, the lipid layer dissociates, facilitating the simultaneous release of PTX and Gem [119]. In this platform, MSNs offer exceptionally high drug-loading capacity, while the lipid coating imparts dual pH- and thermo-responsive release capability that significantly reduces the risk of premature leakage in systemic circulation relative to single pH-responsive systems. Trastuzumab conjugation further ensures active targeting of HER2-overexpressing cells. However, this platform also faces limitations: the slow biodegradation rate of MSNs necessitates systematic evaluation of long-term accumulation risks, and the complex structure of L-MSNs markedly increases preparation complexity and batch-to-batch quality control challenges, thereby posing obstacles to large-scale manufacturing.

##### VEGF

Unlike trastuzumab and tmTNF-α mAb, VEGF antibodies can specifically target endothelial cells involved in the neovascularization of solid tumors, effectively blocking the VEGF signaling pathway to inhibit angiogenesis. This broadens their applicability, albeit with potentially lower targeting efficiency than direct tumor-cell-targeting strategies. Sanyog Jain developed pH-sensitive PEGylated liposomes that are functionalized with VEGF antibodies for the delivery of DTX. This carrier demonstrated an approximately 3.17-fold enhancement in drug uptake in MCF-7 cells and significantly amplified both pro-apoptotic and anti-angiogenic activities [120]. Given the widespread clinical use of liposomes and the FDA approval of the VEGF antibody bevacizumab for multiple solid tumors, this platform holds considerable translational potential. However, achieving batch-to-batch consistency in large-scale production and maintaining the stability of pH-sensitive liposomes under physiological pH conditions remain key challenges.

##### CD326

CD326 (EpCAM) is aberrantly overexpressed on the surface of most malignant tumors derived from epithelial tissues, as well as on tumor stem cells. Compared with the aforementioned HER2-targeted, tmTNF-α-targeted, and VEGF-antibody-targeted strategies, this approach can simultaneously eliminate both differentiated tumor cells and the self-renewing CSC subpopulation, thereby reducing the risk of recurrence and metastasis. Li et al. engineered polymeric NPs that are crosslinked with a CUR derivative, incorporating glutathione (GSH)-responsive crosslinks to stabilize the carrier structure. They further modified these NPs with CD326 antibodies to facilitate active targeting [121]. However, its low-level expression in normal epithelial tissues may lead to off-target toxicity in normal organs such as the gastrointestinal tract and liver. Moreover, unlike trastuzumab and the VEGF antibody, which are supported by mature clinical data, the application of the CD326 antibody in nanocarriers remains at the preclinical stage, with extremely limited data on immunogenicity and long-term safety.

#### 5.2.4. Aptamer-Modified NDDS

Aptamers are short single-stranded DNA or RNA sequences obtained through in vitro selection methods such as systematic evolution of ligands by exponential enrichment (SELEX). They can specifically recognize target molecules, functioning similarly to antibodies but are nucleic acids in nature. Esmaeili et al. engineered a MUC-1aptamer-modified nanoplatform based on graphene oxide quantum dots (GOQD), chitosan, and polyethylene glycol for BC diagnosis and therapy integration. This platform combines pH-responsive drug release, MUC-1-specific recognition, fluorescence sensing, and imaging capabilities. Its stem-loop structure quenches GOQD fluorescence through energy transfer mediated by C-G base pairing. Upon binding to MUC-1 and subsequent dissociation in the acidic conditions of the tumor microenvironment, fluorescence is restored, simultaneously triggering drug release to achieve a diagnostic–therapeutic synergy [122]. Compared with antibodies, aptamers offer low chemical synthesis cost and good batch-to-batch reproducibility, and their nucleic acid nature confers low immunogenicity, thereby mitigating the risks associated with repeated administration. In addition, the GOQD core enables real-time monitoring of drug delivery and tumor response, a capability that is difficult to achieve with liposomal or polymeric carriers. Nevertheless, several major hurdles remain before clinical translation of this platform, particularly regarding stability, safety, and manufacturing consistency. For instance, aptamers are susceptible to rapid nuclease degradation, which severely compromises their circulation half-life in vivo. In addition, carbon-based nanomaterials may be retained long-term in organs, raising concerns over chronic accumulation.

#### 5.2.5. Small Molecule-Modified NDDS

Hyaluronic acid (HA)-mediated nanodelivery systems significantly enhance drug accumulation at tumor sites while reducing toxic side effects by specifically targeting the CD44 receptor, which is highly expressed on the surfaces of tumor cells. HA is a natural endogenous polysaccharide with excellent biocompatibility and biodegradability, and is much less expensive than antibodies. However, basal expression of CD44 in normal tissues (e.g., liver and immune cells) may cause nonspecific distribution. For instance, Sharma et al. covalently linked HA to ursolic acid to construct amphiphilic prodrug NPs. The co-loading of these NPs with PTX facilitated enzyme-responsive release, thereby enhancing cellular uptake, cytotoxicity, and pro-apoptotic capacity [123]. Moreover, various natural products, such as CUR [124] and berberine [125], have been successfully loaded onto HA-modified nanocarriers, demonstrating synergistic effects that include the reversal of drug resistance and suppression of metastasis, alongside efficient targeted delivery. Although the dual-ligand combination (HA and FA) improves targeting specificity, it simultaneously increases manufacturing complexity and hampers batch-to-batch quality control. Furthermore, unlike the single natural active ingredient used by Sharma and Paoletti, berberine derivatives require chemical modification, specifically the incorporation of a 16-carbon aliphatic chain, which adds additional synthetic steps. In contrast to the chemotherapy-dependent approach of Lin’s platform, the present platform harnesses berberine’s intrinsic photothermal properties and IDO-1 inhibitory activity to establish a chemotherapy-free immunotherapeutic modality. By concurrently inhibiting IDO-1 and blocking PD-L1, it mitigates both tryptophan catabolic suppression and T-cell exhaustion, thereby providing a systematic route for converting immunologically “cold” tumors into “hot” ones [126]. Overall, HA-based active targeting systems exhibit multiple advantages in BC therapy, including synergistic enhancement, reduced toxicity, and the ability to overcome drug resistance.

Folic acid (FA)-based nanosystems can actively target tumor cells that overexpress FA receptors, thereby enhancing drug delivery efficiency due to their low immunogenicity, ease of modification, and high stability. This system not only facilitates tumor-specific delivery but also overcomes drug resistance and improves therapeutic efficacy. For example, FA-mediated nanohybrid systems enable the co-delivery of drugs and genes, effectively reversing multidrug resistance by silencing P-gp proteins [127]. Additionally, FA-modified drug-loaded NPs promote tumor cell apoptosis through mechanisms such as inducing mitochondrial calcium overload. These advancements underscore the significant value of FA-targeting strategies in cancer therapy [128]. The FA-targeting strategy offers inherent translational advantages, including facile synthesis and batch-to-batch consistency of small-molecule ligands. However, the selectivity of folate receptor expression and potential interference from ligand competition require careful consideration in preclinical evaluations.

NPs functionalized with progesterone (PR) as a ligand can achieve active targeting by exploiting the high expression of progesterone receptors on the surfaces of tumor cells. This strategy significantly enhances drug accumulation and uptake at tumor sites, thereby improving antitumor efficacy. For example, PR-modified casein-calcium ferrate nanocomposites can effectively carry naringin, facilitating pH-responsive release while substantially reducing the drug’s half-maximal inhibitory concentration (IC50) [129]. NPs functionalized with 5-boronopicolinic acid (BA) serve as targeted ligands that specifically recognize sialic acid (SA) overexpressed on the surfaces of tumor cells. This specificity significantly enhances the accumulation of NPs at tumor sites and promotes cellular uptake, ultimately improving antitumor efficacy [130]. PR-targeted delivery is limited by hormone receptor status, rendering it unsuitable for hormone receptor-negative subtypes such as TNBC. The core advantage of the BA targeting strategy lies in its universality; however, off-target effects on normal tissues and the safety of boronic acid metabolism must be prioritized for clinical translation.

#### 5.2.6. Clinical Challenges of Active Targeted NDDS

Although ligand-mediated active targeting has demonstrated significant success in vitro, its clinical translation remains hindered by multiple obstacles. The primary challenge is receptor heterogeneity. Significant variations in target receptor expression exist within the same tumor tissue as well as between primary and metastatic lesions, frequently leading to the evasion of cellular subpopulations lacking the targeted receptors. Furthermore, the basal expression of targeted receptors in healthy tissues induces off-target accumulation. Meanwhile, the protein corona formed in the bloodstream not only severely masks targeting ligands but also accelerates the non-specific clearance of the delivery system. Beyond the binding-site barrier and restricted interstitial penetration detailed previously, significant biological heterogeneity among individual patients severely undermines the universal applicability of active targeting strategies. Furthermore, the clinical translation of actively targeted nanomedicines faces macroscopic challenges such as scalable manufacturing and regulatory hurdles, which will be discussed in subsequent sections.

**Table 4:** Some of the natural product-based NDDS through active targeting mechanisms proposed for BC.

Modified Ligands	Nanodelivery System	Natural Products (Co-Loaded)	Size (nm)	Drug Loading (%)	Experimental Design	Key Findings	Ref
Peptide (iRGD)	iRGD-guided Silica/Gold Nanoparticles	PTX	160	23.17 ± 0.14	In vitro (MDA-MB-231 cells) In vivo (Xenograft: athymic nude female mice, MDA-MB-231 cells)	Inhibits the rapid growth of tumor cells and promotes tumor cell apoptosis	[105]
	PEG- and iRGD-modified MoS_2_ nanoparticles	CPT-11 (Fe^2+^ ions)	259.6	25.6	In vitro (4T1 cells) In vivo (4T1 tumor-bearing mice)	Increases intracellular H_2_O_2_ content and activates ferroptosis	[106]
Peptide (cRGD)	cRGD- and HA-modified Prussian blue nanoparticles	CPT prodrug copolymer	~151.4	9.2 ± 0.3	In vitro (4T1 cells) In vivo (4T1 tumor-bearing mice)	Prolongs blood circulation, enhances tumor accumulation, and downregulates the expression of HSP70	[107]
Peptide (cRGDfK)	Sulfo-cyanine 5.5 (Cy5.5)-conjugated and cRGDfK-incorporated PEG-PLGA-based polymeric nanoparticles	PTX (CUR)	162.9~182.9	PTX: 2.25 ± 0.3 CUR:11.76 ± 0.7	In vitro (4T1 cells) In vivo (Xenograft: BALB/c mice, 4T1 cells)	Provides a clear picture of biodistribution and improves drug accumulation at the tumor site	[108]
Peptide (R9dGR)	R9dGR-modified nanostructured lipid carriers (NLCs)	PTX (Gambogic acid)	22.03 ± 0.48	PTX: 6.27 ± 0.04 GA:7.55 ± 0.06	In vitro (4T1, MDA-MB-231, and MCF-7 cells) In vivo (Xenograft: nude mice, 4T1 cells)	Overcomes the multidrug resistance of PTX and inhibits tumor cell migration and invasion	[109]
Peptide (CREKA)	CREKA peptide-modified nanodiscs	CPT	15.7 ± 0.3	NA	In vitro (HUVEC, 4T1 cells) In vivo (4T1 tumor-bearing mice)	Activates the coagulation cascade to amplify tumor-specific drug delivery	[110]
Peptide (SILY)	SILY-modified NLCs within a shell of pNIPAM	PTX	380.4 ± 4.4	NA	In vitro (MCF-7, T-47D and MCF-10A cells)	Improves the effectiveness of the treatment and reduces damage to healthy tissue	[111]
Peptide (LHRH)	LHRH-targeted disulfide crosslinked micelles	PTX	28.1 ± 2.8	NA	In vitro (MDA-MB-231 and MDA-MB-435 cells) In vivo (Xenograft: BALB/c mice, MDA-MB-231cells; Xenograft: NSG mice, BR0620F; Transgenic murine mammary carcinoma mice	Facilitates the tumor distribution and penetration of payloads	[112]
Peptide (PDL1 binding peptide)	Human serum albumin nanoparticles functionalized with PDL1-binding peptides	CUR	246.5	5.52	In vitro (MDA-MB-231, SK-BR-3, and MCF-7 cells)	Exhibits higher cell internalization, cytotoxicity, and apoptosis rates in MDA-MB-231 cells	[114]
Peptide (LyP1)	LyP1-functionalized regenerated silk fibroin-based nanoparticles	Quercetin	203.2	5.6	In vitro (4T1 cells) In vivo (Xenograft: BALB/c mice, 4 T1 cells)	Promotes apoptosis and inhibits migration and invasion	[115]
Protein (Transferrin)	MOF coated with transferrin-decorated pH-sensitive lipid layer	Piperlongumine	185 ± 5.7	12.3 ± 4.33%	In vitro (4T1 cells) In vivo (Xenograft: BALB/c mice, 4 T1 cells)	Efficiently kills tumor cells by activating ferroptosis and pyroptosis pathways	[116]
Antibody (tmTNF-α)	PTX nanoparticles conjugated with tmTNF-α monoclonal antibody	PTX	242.5	16.9 ± 0.38	In vitro (MCF-10A, MDA-MB-453 and MDA-MB-231 cells) In vivo (Xenograft: BALB/c mice, MDA-MB-231 cells)	Promotes apoptosis, regulates MAPK, PI3K/AKT/mTOR, and AMPK/NF-κB pathway and inhibits the epithelial–mesenchymal transition process of TNBC	[118]
Antibody (Trastuzumab)	Trastuzumab-conjugated dual stimuli lipid-coated mesoporous silica nanoparticles	PTX (Gemcitabine)	200	PTX: 26.7 GEM: 38.8	In vitro (SK-BR-3 cells)	Increases release of PTX and GEM, specifically binds to more HER2+ breast cancers and inhibits their growth	[119]
Antibody (VEGF)	VEGF antibody-functionalized PEGylated pH- sensitive liposomes	DTX	206.77 ± 28.8	5	In vitro (MCF-7 cells) In vivo (Induced: Sprague–Dawley rats, DMBA)	Inhibits tumor growth and metastasis by inhibiting tumor angiogenesis	[120]
Antibody (CD326)	Monoclonal antibody CD326-modified nanoparticles, stimulated by GSH	CUR	110	10.9	In vitro (MCF-7 cells) In vivo (MCF-7 cell xenograft mouse model)	Shows good inhibition and apoptosis-promoting effects on MCF-7 cells	[121]
Aptamer (MUC-1)	GOQD chitosan PEG nanoconjugate bound to MUC-1 aptamer	CUR	150	NA	In vitro (MCF-7 breast cancer cells and HT-29 colon cancer cells)	Exhibits good blood compatibility, higher cytotoxicity in MCF-7 cells	[122]
Small molecule (HA)	CD44-targeted enzyme-sensitive nanoparticles	PTX(Ursolic acid)	173	11.58	In vitro (MDA-MB-231 and 4T1 cells) In vivo (Xenograft: BALB/c mice, 4T1 cells)	Enhances cellular uptake and synergistic antitumor effect	[123]
	Soft nanoparticles or nanogels spontaneously assembled by HA and estradiol	DTX (Curcumin)	342 ± 23	4.0 ± 0.1	In vitro (MCF-7 cells)	Inhibits the growth of ER + breast cancer cells	[124]
	DSPE-PEG-FA- and HA-modified nanodrugs	9-O octadecyl-substituted berberine derivatives (Doxorubicin)	213.3 ± 3.0	68.17 ± 0.04	In vitro (MDA-MB-231 cells) In vivo(xenograft: nude mice, MDA-MB-231 cells)	Inhibits mitochondrial membrane potential, increases the production of reactive oxygen species, activates the caspase cascade, and decreases MMP-2 and MMP-9 activity	[125]
	HA-coated self-assembly active nano modulators	Berberine (ICG)	174.9	BBR: 63.7 ICG: 21.5	In vitro (4T1 cells) In vivo (4T1 tumor-bearing mice)	Downregulates PD-L1 and IDO-1 protein expression, induces increased apoptosis, and prevents tumor metastasis	[126]
	CD44 and biotin receptors dual- targeted enzyme-sensitive HA nanogel	PTX	149.1 ± 1.6	15.28 ± 0.10	In vitro (4T1 cells) In vivo (Xenograft: BALB/c mice, 4T1 cells)	Exhibits excellent tumor-targeting and a better pharmacokinetic profile	[131]
	MSN combined with HA	Curcumin	161.3	14.76	In vitro (MDA-MB-231 cells, MCF-7 cells) In vivo (Swiss albino mice carrying EAC solid tumors)	Blocks cell cycle, inhibits cell migration, induces mitochondrial-dependent apoptosis	[132]
	Amphiphilic HA nano-micelles	Quercetin	99.2	10.6	In vitro (MCF-7 and HL7702 cells) In vivo (MCF-7 tumor-bearing mice)	Improves the targeting efficiency, reduces the toxicity, and inhibits the growth of breast cancer cells	[133]
	Mineralized HA-SS-tetradecyl nano-carrier	Sulforaphane	85.90 ± 3.49	33.64 ± 1.33	In vitro (MDA-MB-231, Hs578T, MCF-7 and MCF10A cells) In vivo (Xenograft: BALB/c nude mice, MDA-MB-231 cells)	Inhibits tumor growth and the self-renewal ability of breast cancer stem cells	[134]
	HA-modified Pluronic^®^ mixed copolymer nanoparticles	Thymoquinone	19.3 ± 3.2	NA	In vitro (MDA-MB-231 cells, MDA-MB-468 cells and 4T1 cells) In vivo (Xenograft: chick embryo, MDA-MB-231 cells; 4T1 tumor-bearing BALB/c mice)	Upregulates microRNA-361, downregulates Rac1- and RhoA-mediated cell migration, reduces the secretion of VEGF-A	[135]
ChS	ChS-coated MSNs	QC (PTX)	~227.2	PTX: 5.29 ± 0.38 QC: 5.13 ± 0.19	In vitro (MCF-7/ADR cells) In vivo (4T1 tumor-bearing BALB/c mice)	Reduces the expression of P-glycoprotein, inhibits the efflux of PTX, leads to cell cycle arrest at the G2/M phase, microtubule destruction, and increases the apoptosis rate	[136]
FA	FA-mediated, CS-grafted disulfide-containing PEI copolymer-based silica nanohybrids	PTX	138.7 ± 10.7	7.43 ± 0.29	In vitro (MCF-7 cells, MCF-7/ADR cells)	Induces apoptosis in drug-resistant breast cancer cells and reverses resistance to PTX	[127]
	MNPs stabilized with PEG and modified with FA	CUR	6~20	NA	In vitro (MCF-7 cells, A549 cells)	Exhibits stronger cytotoxicity and better internalization	[137]
	FA-coupled chitosan-functionalized protamine and dextran	CUR	22~80	NA	In vitro (MCF-7 cells)	Induces apoptosis and further internalization	[138]
	FA-functionalized carboxymethyl chitosan/calcium phosphate hybrid nanoparticles	CUR	179	40.7	In vitro (MCF-10A cells, MDA-MB-231 cells and 4T1 cells) In vivo (4T1 tumor-bearing mice)	Triggers the mitochondrial apoptosis pathway by mitochondrial calcium overload	[128]
	FA- and PEG-modified ZIF-8	Baicalin	176 ± 8.1	41.45 ± 1.43	In vitro (MCF-7 cells) In vivo (4T1 inoculated female BALB/c mice)	Better uptake of nanoparticles by tumor cells and thus better killing of cancer cells	[139]
PR	PR-targeted magnetic CaFe_2_O_4_ nanohybrid	Hesperidin	146	NA	In vitro (L929, MDA-MB-231 and SKOV-3 cells)	Reduces the semi-inhibitory concentration of the drug and enhances the anticancer activity	[129]
BA	BA-modified PLGA-PEI nanoparticles	Camptothecin	120~150	3.3	In vitro (4T1, MCF-7 and EA.hy926 cells) In vivo (4T1 tumor-bearing mice)	Improves drug delivery efficiency and achieves good anti-tumor effects	[130]

Notes: NA, no data available; pNIPAM, poly(N-isopropylacrylamide); NLCs, nanostructured lipid carriers; MOF, metal–organic framework; PL, piperlongumine; CUR, curcumin; MSN, mesoporous silica nanoparticles; HA, hyaluronic acid.

### 5.3. Stimuli-Responsive Targeted NDDS

To overcome complex physical and biological barriers, evade the binding-site barrier, and achieve lysosomal escape, stimuli-responsive targeted NDDSs have emerged as an indispensable delivery paradigm [140]. Researchers have exploited the pathological hallmarks of the TME, such as low pH, hypoxia, elevated GSH levels, and specific enzyme overexpression, to develop internal stimuli-responsive NDDSs. By utilizing TME abnormalities as natural triggers, these systems undergo profound physicochemical transformations like charge reversal or size shrinkage [141]. Such dynamic changes enable the nanocarriers to actively penetrate the dense stroma, efficiently escape endosomes and lysosomes, and precisely release their payloads into the cytoplasm of deep-seated BC cells [142].

However, internal stimuli-responsive NDDSs are frequently limited by the high heterogeneity of the TME among individual patients. To achieve absolute spatiotemporal controllability, external stimuli-responsive NDDSs have been developed to facilitate on-demand drug release using non-invasive physical signals like near-infrared light, ultrasound, or magnetic fields [143]. Furthermore, to overcome the limitations of single-trigger mechanisms, advanced delivery platforms have strategically integrated multiple internal and external stimuli to construct multi-responsive systems, thereby maximizing release controllability and significantly enhancing antitumor efficacy [144]. Table 5 and Table 6 respectively summarize various internal and external stimuli-responsive platforms based on natural products currently evaluated for breast cancer therapy.

#### 5.3.1. Internal Stimuli-Responsive NDDS

##### PH-Responsive NDDS

Tumor tissues and their intracellular microenvironments (pH ≈ 5.0–6.8) exhibit significantly lower pH levels compared to normal physiological pH (≈7.4). pH-responsive NPs utilize this endogenous acidity gradient to facilitate targeted drug delivery within the BC microenvironment. These systems are typically constructed from materials that contain pH-responsive functional groups, such as carboxyl, amino residues, or imine bonds. Upon reaching the tumor site, the acidic environment induces charge reversal, conformational changes, or chemical bond cleavage, thereby enabling precise drug release [145]. Additionally, the zeolitic imidazolate framework-8 (ZIF-8) can release reducing agents through an acid-triggered structural collapse, which accelerates the reductive cleavage of disulfide bonds. This process leads to the rapid disintegration of the outer polymer shell, ultimately facilitating swift drug release [146]. Furthermore, the phenolic hydroxyl group of the CUR molecule interacts with the terminal alkyne group of the polymer, resulting in the formation of a vinyl ether bond. This bond is capable of responding to pH changes, facilitating the release of drugs and inducing immunogenic cell death in tumor cells [147]. Although pH-responsive NDDS represent a prominent technology in tumor therapy, their responsive behavior is influenced by multiple factors, including particle size, morphology, and surface properties. Further systematic research is necessary to achieve more precise control over drug release.

##### Redox Responsive NDDS

Redox-responsive NPs facilitate targeted drug delivery by exploiting the reducing microenvironment characterized by elevated concentrations of GSH within tumor cells [148]. High levels of GSH can cleave responsive groups, such as disulfide bonds, borate esters, and thioethers, in the NP carrier, thereby triggering carrier dissociation and enabling rapid drug release. Lou et al. linked PTX to an albumin-binding ligand via a disulfide bond, utilizing albumin as a carrier to achieve prolonged circulation and active targeting [149]. Similarly, Hao et al. connected PTX to a copper ion chelator through a disulfide bond, thereby integrating disulfide-responsive drug release with the regulation of copper metabolism [150]. Compared with traditional disulfide bonds, cyclic diselenides exhibit more sensitive response-activation capabilities under high levels of GSH within the tumor microenvironment [151]. These systems cleverly exploit the highly reduced environment found within tumor cells to facilitate intelligent and selective drug release via disulfide bonds. However, disulfide bonds may be prematurely cleaved by trace reductive substances in the bloodstream (such as plasma glutathione), leading to premature drug release and increased systemic toxicity.

##### Enzyme-Responsive NDDS

Enzyme-responsive NDDS facilitate targeted accumulation and controlled drug release by integrating peptide chains or chemical bonds specifically designed for recognition and cleavage by enzymes that are highly expressed in the tumor microenvironment. Upon reaching the tumor site, enzymatic activation instigates the structural dissociation of the carrier, induces surface charge reversal, or cleaves drug bonds, thereby enhancing both targeting specificity and release efficiency. Sharma R developed NPs utilizing a HA scaffold. Upon reaching the tumor site, these NPs undergo hydrolysis facilitated by the highly expressed enzyme hyaluronidase, leading to structural disintegration and an accelerated release of PTX. Concurrently, HA facilitates tumor targeting through its binding to the CD44 receptor [123]. Building upon the response of the HA enzyme, Gao et al. introduced an additional lipase-responsive chemical bond. This innovation established a dual-enzyme synergistic response mechanism that further enhances the precision and efficiency of drug release [131]. However, the expression of targeted enzymes within the tumor microenvironment demonstrates spatial heterogeneity and variability between patients, which may result in inconsistent drug responses, inadequate release, or premature release of therapeutic agents. Moreover, specific targeted enzymes, such as matrix metalloproteinases, may be upregulated under inflammation, leading to off-target release and associated side effects.

##### Dual and Triple Stimuli-Responsive NDDS

The elements of the tumor microenvironment are interconnected rather than evolving independently. Therefore, the development of multi-stimulus responsive nanocarriers that integrate multiple response mechanisms presents two significant advantages: the triggered release of medication and enhanced therapeutic efficacy.

Currently, dual-stimulus-responsive nanodelivery systems have emerged as a prominent research focus in the field of tumor-targeted drug delivery. Various NPs employ distinct delivery strategies to address tumor heterogeneity and dynamic microenvironments, emphasizing specific therapeutic mechanisms. For example, the lipid-coated mesoporous silica nanoparticles (L-MSNs) developed by Nasri N et al. utilize the acidic tumor microenvironment and mild hyperthermia to trigger the release of PTX and gemcitabine (Gem). Their surface is modified with trastuzumab for specific targeting of HER2-positive BC [119]. Moreover, a carrier-free nano-cocktail based on prodrug self-assembly, featuring a precise ratio of docetaxel and salinomycin, not only effectively eradicates bulk breast cancer cells but also significantly inhibits the enrichment of cancer stem cells (CSCs), demonstrating prominent advantages in tumor eradication and metastasis prevention [152]. Another study developed a pH/redox dual-responsive nanocarrier that effectively releases PTX under acidic conditions and delivers small hairpin RNA (shRNA) in the cytoplasm with elevated GSH concentrations. This approach synergistically induces tumor cell death while simultaneously downregulating the expression of the drug resistance protein P-gp, thereby reversing multidrug resistance in BC [127].

Furthermore, triple-responsive systems (e.g., responding to pH, GSH, and light/temperature) integrate multiple release mechanisms to further enhance tumor-targeted accumulation and synergistic therapeutic effects, demonstrating potential for integrated diagnosis and therapy [153]. However, existing research predominantly focuses on chemical drugs. Multi-responsive delivery systems for natural products still face challenges such as structural complexity and loading difficulties, representing a key direction for future design.

**Table 5:** Some of the natural product-based NDDS with internal stimuli-responsive targeting mechanisms proposed for BC.

Stimuli	Sensitivity Principle	Nanodelivery System	Natural Products (Co-Loaded)	Size (nm)	Drug Loading (%)	Experimental Design	Key Findings	Ref
pH value	Ionization or hydrolysis of acid- based groups	Dodecyl groups and diethylethanolamine surface groups modified cationic polyamidoamine dendrimers	PTX	182.5 ± 0.9	96	In vitro (MDA-MB-231, RAW 264.7 and THP-1 cells) In vivo (Xenograft: BALB/c mice, 4T1 cells; Xenograft: NSG mice, MDA-MB-231 cells)	Reduces chemotherapy-induced inflammation and tumor metastases	[154]
		N-O type zwitterionic polymers	CUR (ginsenoside Rg3)	82.66 ± 4.27	CUR: 14.15 ± 2.33 Rg3: 2.26 ± 1.42	In vitro (4T1 cells) In vivo (4T1 tumor-bearing mice)	Induces more immunogenic cell death of tumor cells, reduces the expression of PD-L1, and reduces the amount of cancer stem cells	[147]
		Bicompartmental Janus nanoparticles	PTX (Lapatinib)	175	-	In vitro (BT-474 and MDA-MB-231 cells)	Increases activity against HER2^+^ breast cancer cells and triple-negative breast cancer cells	[155]
		VEGF antibody-functionalized PEGylated pH-sensitive liposomes	DTX	206.77 ± 28.8	5	In vitro (MCF-7 cells) In vivo (Induced: Sprague–Dawley rats, DMBA)	Inhibits tumor growth and metastasis by inhibiting tumor angiogenesis	[120]
		Lipoplex	DTX (sirtuin 1 shRNA)	200	-	In vitro (MDA-MB-231 cells, MCF-7 cells) In vivo (Induced: Sprague–Dawley rats, DMBA)	Increases the efficacy and decreases the toxicity	[156]
		Chitosan microspheres	CUR	11.405	53.60	In vitro (MDA-MB-231 cells)	Inhibits BC cell proliferation	[157]
GSH concentration	Cleavage of disulfide bond	Albumin-bound nanoparticle	PTX	185.5 ± 10.3	-	In vitro (4T1 cells) In vivo (Xenograft: BALB/c mice, 4T1 cells)	Achieves low immunogenicity and great intra-tumor accumulation	[149]
		Nanoparticle	PTX (Copper chelator)	142.4 ± 3.2	24.35 ± 0.36	In vitro (4T1 cells and L929 cells) In vivo (Xenograft: BALB/c mice, 4T1 cells)	Increases oxidative stress- and abnormal metabolism-induced cell death through intracellular copper depletion	[150]
Enzyme expression level	Enzymatic cleavage	CD44-targeted enzyme-sensitive nanoparticles	PTX (Ursolic acid)	173	11.58	In vitro (MDA-MB-231 and 4T1 cells) In vivo (Xenograft: BALB/c mice, 4T1 cells)	Enhances cellular uptake and synergistic antitumor activity	[123]
		CD44 and biotin receptors dual- targeted enzyme-sensitive HA nanogel	PTX	149.1 ± 1.6	15.28 ± 0.10	In vitro (4T1 cells) In vivo (Xenograft: BALB/c mice, 4T1 cells)	Exhibits excellent tumor-targeting and a better pharmacokinetic profile	[131]
Multiple factors	Joint influence of multiple factors	Trastuzumab-conjugated dual stimuli lipid-coated mesoporous silica nanoparticles	PTX (Gemcitabine)	200	PTX: 26.7 GEM:38.8	In vitro (SK-BR-3 cells)	Increases release of PTX and GEM, specifically binds to more HER2^+^ breast cancers, and inhibits their growth	[119]
		FA-mediated, CS-grafted disulfide-containing PEI copolymer-based silica nanohybrids	PTX	138.7 ± 10.7	7.43 ± 0.29	In vitro (MCF-7 cells, MCF-7/ADR cells)	Induces apoptosis in drug-resistant breast cancer cells and reverses resistance to PTX	[127]

#### 5.3.2. External Stimuli-Responsive NDDS

##### Light-Responsive NDDS

Light-responsive NDDS plays a central role in tumor phototherapy, including both PDT and PTT, by enabling precise treatment through activation via near-infrared light. The design of these NDDSs primarily encompasses two major directions. First, in PDT, NPs utilize photosensitizers (PS) to produce ROS upon irradiation with near-infrared (NIR) light, leading to tumor cell death. For example, the biocompatible photosensitive derivative developed by the Sun team covalently conjugates a photosensitizer, pyro-de-magnesium chlorophyll-a (PPA), with diphenylalanine, generating ROS upon 660 nm laser irradiation while simultaneously activating a prodrug N-CPT in response to the resulting hypoxic microenvironment. This dual action achieves synergistic effects in chemotherapy [158]. As research progresses, researchers have shifted their strategies for optimizing drug delivery from targeting tumor cells to targeting the vascular systems upon which tumors depend for survival. Building on this approach, Yang et al. designed a two-component nanosystem that first induces vascular embolism using photosensitive NPs to obstruct blood supply. Subsequently, it employs endogenous signals at the embolized site to capture CPT-loaded nanoplatforms, thereby achieving targeted vascular delivery [110]. However, the dense tumor stroma continues to impede drug penetration. To address this challenge, Feng et al. developed a multistage responsive nanoplatform that first releases the matrix-degrading drug cyclopamine to enhance deep tissue penetration. This is followed by the rapid release of photosensitizer Ce6 triggered by intracellular reduction, ultimately achieving combined antitumor effects [159].

In contrast, PTT employs photothermal agents such as gold nanorods and graphene oxide to convert near-infrared light into heat, thereby facilitating the thermal ablation of tumors. However, PTT faces three major challenges: thermal tolerance, shallow penetration, and limited therapeutic efficacy. To address the heat tolerance issues associated with PTT, the Yang laboratory developed targeted Prussian blue NPs loaded with a camptothecin prodrug that suppresses the upregulation of heat shock protein 70 (HSP 70) induced by PTT, thereby enhancing PTT sensitivity [107]. Meanwhile, Shen’s team developed a ZIF-8 loaded with liquid metal NP that releases CUR in acidic microenvironments to activate autophagy, thereby overcoming thermal tolerance and downregulating PD-L1 [160]. Compared with conventional PTT, low-temperature PTT can also reduce thermal damage to normal tissues and lower the risk of tumors developing thermal resistance. Li et al. designed a nanoplatform that systematically eliminates heat resistance potential by inducing ferroptosis, cell cycle arrest, and actively degrading HSP70 [83].

To address the issue of insufficient tissue penetration, Gao et al. developed a light-responsive injectable hydrogel composed of polydopamine. This innovative hydrogel facilitates photothermal ablation and enables on-demand drug delivery [161]. Notably, PTT can also induce immunogenic cell death to activate systemic immunity. For instance, Sun’s team demonstrated that their self-assembled NPs, when combined with PD-L1 antibodies, not only suppress primary tumors and lung metastases but also enhance T-cell infiltration [126]. In addition to regulating HSP 70 and combining with chemotherapy or immunotherapy to address the limitations of PTT, exploring deep synergies between PTT and novel killing mechanisms, such as the disruption of cellular ion homeostasis, represents a crucial direction for achieving significant breakthroughs. Wang et al. combined PTT with a mitochondrial Ca^2+^ overload strategy to develop a multimodal Ca^2+^ nano-modulator. This modulator induces tumor cell apoptosis through a dual-mode synergistic action of ionic interference and photothermal effects [162]. By integrating immune-enhancing natural products with photoactivatable gene-silencing technologies (such as RRM2 siRNA) via nanoplatforms, it is promising to achieve more potent synergistic immunotherapy for breast cancer [163]. Despite the exceptional therapeutic gain provided by light-responsive nanodelivery systems, clinical translation still faces challenges such as limited tissue penetration depth and the biosafety of photosensitizers. Future research should focus on developing more biocompatible natural photoactive carriers and exploring cascaded linkages with endogenous stimuli-responsive mechanisms to achieve deeper, more efficient, and safer targeted therapy for breast cancer.

##### Magnetic Responsive NDDS

Magnetically stimulated responsive materials (with magnetic NPs at their core) demonstrate significant potential in deep-tissue tumor therapy due to their deep tissue penetration capabilities. First, the targeted delivery capability of magnetic NPs is crucial for enhancing local drug accumulation. For instance, chitosan-coated iron oxide NPs loaded with CUR derivatives, which significantly improved cellular uptake and toxicity when subjected to an applied magnetic field [164]. Secondly, magnetic responsiveness can be integrated with intelligent responses to the tumor microenvironment to facilitate on-demand drug release. For example, the progesterone-targeted magnetic casein-ferroferrite nanohybrids, which are loaded with naringin and developed by Purushothaman et al., demonstrate pH-responsive drug release properties [129]. Additionally, magnetic response systems can be utilized to overcome tumor resistance and facilitate synergistic therapy. For example, salinomycin-loaded polypropylene sulfide-coated magnetic NPs, which are responsive to the tumor microenvironment, can effectively reverse multidrug resistance and synergistically induce cell death when used in conjunction with chemotherapy [165]. Currently, magnetoresponsive nanosystems are evolving toward multi-functional integration and streamlined fabrication. In addition to conventional organic polymers, magnetic platforms based on inorganic biomaterials, such as calcium phosphate, have garnered significant attention due to their excellent biocompatibility [166]. Similarly, the magnetic plasma-based drug-loaded nanocapsules proposed by Fluksman et al. combine magnetic targeting with photothermal activation, thereby enabling enhanced treatment at low doses through multiple physical stimuli [167]. In summary, magnetic-responsive nanocarriers have evolved into intelligent therapeutic platforms that enable stimulus-triggered drug release, durable MDR reversal, and multimodal synergy. Future work should integrate imaging or immunomodulatory functions to accelerate clinical translation.

##### Ultrasound Responsive NDDS

Magnetic triggers are constrained by inherent limitations, including high costs and low spatial focusing accuracy. In contrast, ultrasound technology presents advantages such as lower costs, superior deep tissue penetration, and controllable acoustic cavitation effects. Based on their core response mechanisms, existing systems can be primarily categorized into three types: physically triggered drug-release systems, sonodynamic-activated systems, and synergistic-assisted systems.

First, the physically triggered drug delivery system relies on low-boiling-point phase-change materials (such as perfluoropentane, PFP) undergoing liquid–gas transitions under ultrasound exposure to achieve imaging, localization, and instantaneous drug release. For instance, Zhang et al. designed PLGA-coated PFP NPs that sequentially release anti-miR-221 and PTX upon ultrasonic triggering while disrupting the tumor stroma to enhance penetration [168]. In addition, sonodynamic therapy (SDT) employs ultrasound to activate photosensitizers, generating cytotoxic ROS. This method allows for deep tissue penetration while sparing surrounding healthy tissues. The efficacy of SDT is contingent upon high-performance photosensitizers, such as zirconium-based porphyrin metal–organic frameworks (PCN-222) and the mitochondria-targeted TPP-Ce6. Additionally, co-loading pro-oxidative drugs like piperazine enhances tumor cell sensitivity to oxidative stress [169]. Kang et al. improved SDT efficacy and selectivity through precise mitochondrial targeting [170], while Zhang et al. developed a multifunctional nanosystem that integrates low-intensity focused ultrasound with SDT to inhibit primary BC growth and lung metastasis [171]. Finally, the synergistic delivery system utilizes exogenous microbubbles as core components, leveraging their ultrasonic cavitation effects to enhance drug release and cellular uptake. For instance, Radha R. et al. employed a stepwise administration of CUR liposomes in conjunction with exogenous microbubbles; the ultrasonic triggering of microbubble cavitation simultaneously disrupted liposome structures and increased cell membrane permeability, significantly boosting drug activity and pro-apoptotic effects [172].

**Table 6:** Some of the natural product-based NDDS through external stimuli-responsive targeting mechanisms proposed for BC.

Stimuli	Responsive Element	Nanodelivery System	Natural Products (Co-Loaded)	Size (nm)	Drug Loading (%)	Experimental Design	Key Findings	Ref
Light (PDT)	Pheophorbide-diphenylalanine peptide	Tandem-responsive nanoassemblies	N-CPT	~70	10.4	In vitro (4T1 cells) In vivo (xenograft: BALB/c mice, 4T1 cells	Induces high levels of ROS and inhibits hypoxia-induced tumor metastasis.	[173]
	Nickel-manganese-iron complexes	Riboflavin and HA encapsulated spinel ferrite	CUR	70	11.3 ± 0.6	In vitro (MDA-MB-231 cells)	Increases the cytotoxicity under illumination	[174]
	RGD-modified pyropheo-phorbide-a (Ppa) micelles	CREKA peptide-modified nanodiscs	CPT	15.7 ± 0.3	NA	In vitro (HUVEC, 4T1 cells) In vivo (4T1 tumor-bearing mice)	Activates the coagulation cascade to amplify tumor-specific drug delivery	[110]
	Chlorin e6 (Ce6)	Bovine serum albumin (BSA)-based nanoplatform	CYC (HA-SeSe-Ce6)	35	NA	In vitro (4T1 cells) In vivo (4T1 tumor-bearing mice model)	Targets tumor cell internalization and smart drug release	[159]
	Cy780-Acryl	NIR-nanogels	PTX	110 ± 35	NA	In vitro (4T1 cells) In vivo (Xenograft: BALB/c mice, 4T1 cells)	Inhibits tumor growth and reduces tumor metastasis	[77]
Light (PTT)	Prussian blue nanoparticles	cRGD- and HA-modified Prussian blue nanoparticles	CPT prodrug copolymer	~151.4 nm	9.2 ± 0.3%	In vitro (4T1 cells) In vivo (4T1 tumor-bearing mice)	Prolongs blood circulation, enhances tumor accumulation, and downregulates the expression of HSP70	[107]
	Liquid metal nanoparticle	HA/alendronate-functionalized ZIF-8	CUR	145.76 ± 1.28	14.76	In vitro (RAW 264.7 cells, and MCF-10A cells) In vivo (a bone metastasis model in female BALB/c mice	Inhibits tumor cell progression, migration, and invasion, induces autophagy, and resists heat tolerance	[160]
	MoS_2_ nanoparticles	PEG- and iRGD-modified MoS_2_ nanoparticles	CPT-11 (Fe2^+^ ions)	259.6	25.6	In vitro (4T1 cells) In vivo (4T1 tumor-bearing mice)	Increases intracellular H_2_O_2_ content and activates ferroptosis	[106]
	Polydopamine (PDA)	Sulfhydryl-modified hyaluronic acid (HA-SH) crosslinked with polydopamine (PDA) hydrogel covalently attached to HSA	PTX	409.1	16.6	In vitro (4T1 and MCF-7 cells) In vivo (Xenograft: BALB/c mice,4T1 cells)	Stimulates the immune response by increasing IL-6 and TNF-α, decreases Ki-67 expression, and increases apoptosis	[161]
	Indocyanine green (ICG)	HA-coated self-assembly active nanomodulators	Berberine (ICG)	174.9	BBR: 63.7 ICG: 21.5	In vitro (4T1 cells) In vivo (4T1 tumor-bearing mice)	Downregulates PD-L1 and IDO-1 protein expression, induces increased apoptosis, and prevents tumor metastasis	[126]
	Indocyanine green	SA crosslinked on the surface of calcium carbonate nanoparticles	CUR (ICG)	~175	40.7	In vitro (4T1 cells) In vivo (4T1 tumor-bearing mice)	Reduces mitochondrial membrane potential and ATP production	[162]
Magnetic	Iron oxide nanoparticles	Chitosan-coated iron oxide nanoparticles	CUR	<150 nm	1.72 ± 0.01	In vitro (MDA-MB-231 cells)	Enhances the cellular internalization, cytotoxicity, and apoptosis of BC cells	[164]
	Iron/Silica Semishell	Polylactic-co-glycolic acid (PLGA) nanoparticles partially coated by a nanodome-shaped iron/silica semishell	PTX	150	0.8	In vitro (MDA-MB-231 cells) In vivo (Xenograft: Athymic Nude-Foxn1nu mice, MDA-MB-231cells)	Increases the local drug concentration in the tumor and reduces systemic side effects	[167]
	Polypropylene sulfide (PPS) and magnetic core (MnFe)	PPS-coated magnetic nanoparticles	Salinomycin (SAL)	125	1.9	In vitro (MCF-7 cells, MCF-7 BCSCs)	Reverses multidrug resistance in breast cancer stem cells to achieve long-lasting eradication	[165]
Ultrasound-stimuli-responsive	Perfluoropentane	PLGA nanoparticles	PTX (Perfluoropentane, anti-miR-221 inhibitor)	696	-	In vitro (MDA-MB-231 and 4T1 cells) In vivo (BALB/c nude mice)	Increases the sensitivity of PTX and inhibits cell proliferation in vitro and tumor growth and progression	[168]
	Porphyrinic	Polyethylene glycol (PEG)-coated PCN-222	Piperlongumine (PL)	185.1 ± 8.0	-	In vitro (MCF-7 cells)	Enhances ROS production, thereby triggering cancer-specific apoptosis	[169]
	T-Ce6	Tween 80 micelles	PL	18.28 ± 8.49	-	In vitro (MCF-7 cells)	Increases the generation of ROS, leading to mitochondrial damage and cancer cell apoptosis	[170]
	Ce6	PLGA nanoparticles	DTX(Ce6, Perfluoropentane)	249.5 ± 77.46	NA	In vitro (4T1 cells) In vivo (Xenograft: BALB/c mice, 4T1 cells)	Inhibits the growth of breast cancer tumors and reduces metastatic nodules in the lungs	[171]
	Definity microbubbles	Liposomes	CUR	81.65 ± 2.2	-	In vitro (HCC 1954 cells)	Amplifies apoptosis	[172]

## 6. Subtype-Specific Nanomedicine Strategies for BC

Although the aforementioned targeting and stimuli-responsive delivery strategies constitute universal design principles for nanomedicine, such generalized paradigms struggle to perfectly align with the highly complex molecular heterogeneity of breast cancer. Clinically, this disease predominantly encompasses three major subtypes, including TNBC, HER2-positive BC, and hormone receptor (HR)-positive BC. The molecular driving mechanisms, clinical bottlenecks, and tumor microenvironment (TME) characteristics differ across these distinct subtypes. Therefore, this chapter will comprehensively explore how nanomedical platforms can overcome the limitations of one-size-fits-all designs and implement subtype-specific differentiated designs precisely tailored to the unique pathological vulnerabilities of each subtype.

### 6.1. Alternative Targeting and TME Reprogramming in TNBC

Since TNBC lacks the expression of ER, PR, and HER2 receptors, conventional targeted therapies directed at these receptors are rendered ineffective. Nanodelivery systems effectively overcome this targetless dilemma by utilizing surface modifications with specific alternative ligands such as RGD peptides or HA to guide the precise accumulation of natural products [175]. However, active targeting alone is insufficient to combat the complex biological aggressiveness of this subtype. In the realm of gene therapy, nanocarriers not only overcome the physical barriers of rapid nucleic acid degradation [176] but also synergize deeply with natural products to broadly modulate microRNA expression networks and significantly amplify antitumor activity. Meanwhile, physical combination therapies including PTT and PDT integrated with natural product-loaded nanoplatforms have emerged as major research hotspots due to their non-invasive nature and low susceptibility to drug resistance [63]. More importantly, addressing the inherently immunosuppressive cold tumor characteristics of TNBC by remodeling the tumor microenvironment with nanosystems has become a critical breakthrough strategy. For example, co-delivering chemotherapeutic drugs and immune factors through smart nanosystems can effectively promote dendritic cell maturation and deplete regulatory T cells, thereby comprehensively reversing immunosuppression and activating a systemic antitumor response [177].

In summary, nanomedicine strategies for TNBC are no longer confined to merely compensating for singular target deficiencies but have progressively evolved into a multidimensional therapeutic network that deeply integrates ligand targeting, genetic synergy, physical intervention, and immune remodeling, which provides a promising translational blueprint for overcoming its multiple clinical barriers.

### 6.2. Leveraging Receptor Overexpression for Multi-Level Targeted Delivery in HER2-Positive BC

Unlike TNBC, HER2-positive BC is characterized by HER2 receptor overexpression, a molecular feature widely exploited in ligand-mediated nanodelivery. Although antibody-drug conjugates have achieved clinical success, their drug-to-antibody ratio (DAR) remains restricted by random conjugation chemistry, reaching a strict limit of 2 to 4 because high-DAR species of 6 to 8 suffer from rapid plasma clearance and increased toxicity due to the inherent hydrophobicity of the payloads [178]. Antibody-modified nanoparticles overcome this physical upper limit. Their internal cores can encapsulate an order of magnitude more therapeutic agents and enable multi-drug synergy within a single nanocarrier.

Trastuzumab-modified nanoparticles represent the most classic strategy. They establish a “carrier-intrinsic efficacy” synergistic effect with natural products such as curcumin and resveratrol, demonstrating unique advantages in overcoming trastuzumab resistance [179]. To address the steric hindrance and immune clearance issues caused by full-length antibodies, next-generation platforms are rapidly shifting toward miniaturized ligands such as nucleic acid aptamers, specific peptides, and antibody fragments to achieve deeper tumor penetration. For instance, the HER2 aptamer-functionalized nanoparticles (Apt-HSA-CUR-NPs) developed by Saleh et al. increased curcumin solubility 400-fold and achieved tumor penetration depths significantly superior to those of bulky antibody-modified carriers [180]. Ranging from classic monoclonal antibodies to miniaturized fragments and even bispecific designs, these diversified strategies collectively constitute a multi-level and deeply penetrating targeted delivery matrix specifically tailored for HER2-positive breast cancer.

### 6.3. Reversing Endocrine Resistance in HR-Positive BC

HR-positive BC fundamentally relies on the estrogen receptor signaling axis, establishing endocrine therapy as the standard first-line treatment [181]. However, the emergence of acquired endocrine resistance remains a major clinical bottleneck. Nanomedicine primarily addresses this formidable challenge through two complementary mechanisms encompassing bioavailability enhancement and active resistance reversal. Conventional endocrine drugs frequently encounter critical clinical issues regarding extremely poor absorption and systemic toxicity. By physically encapsulating these traditional agents, nanoparticles not only significantly improve their extremely poor oral absorption efficiency but also achieve precise accumulation within localized tumor sites [182].

In terms of active resistance reversal, nanoplatforms enable the highly efficient co-encapsulation of endocrine drugs such as tamoxifen with specific sensitizing natural products. This combined strategy effectively overcomes the uneven in vivo pharmacokinetic distribution of free individual agents, thereby achieving precise spatiotemporal synchronization and multi-target synergy within the same target cell. For example, Zhang et al. discovered that nanocurcumin significantly mitigates tamoxifen resistance in ER-positive cells by potently inhibiting the PI3K/AKT/mTOR signaling pathway while simultaneously suppressing the epithelial–mesenchymal transition [183].

## 7. Clinical Landscape of Natural Product-Based Nanomedicines in BC

While natural product-based nanomedicines have demonstrated extraordinary therapeutic potential in preclinical murine models, their successful translation into clinical practice for BC remains disproportionately low. Table 7 summarizes recent clinical trials relevant to the clinical applications of NPs. To systematically dissect this gap between preclinical promise and actual clinical viability, the following section analyzes the current clinical landscape categorized by specific formulations and drug delivery platforms.

### 7.1. Clinically Validated Nanomedicines

**Figure 5:**
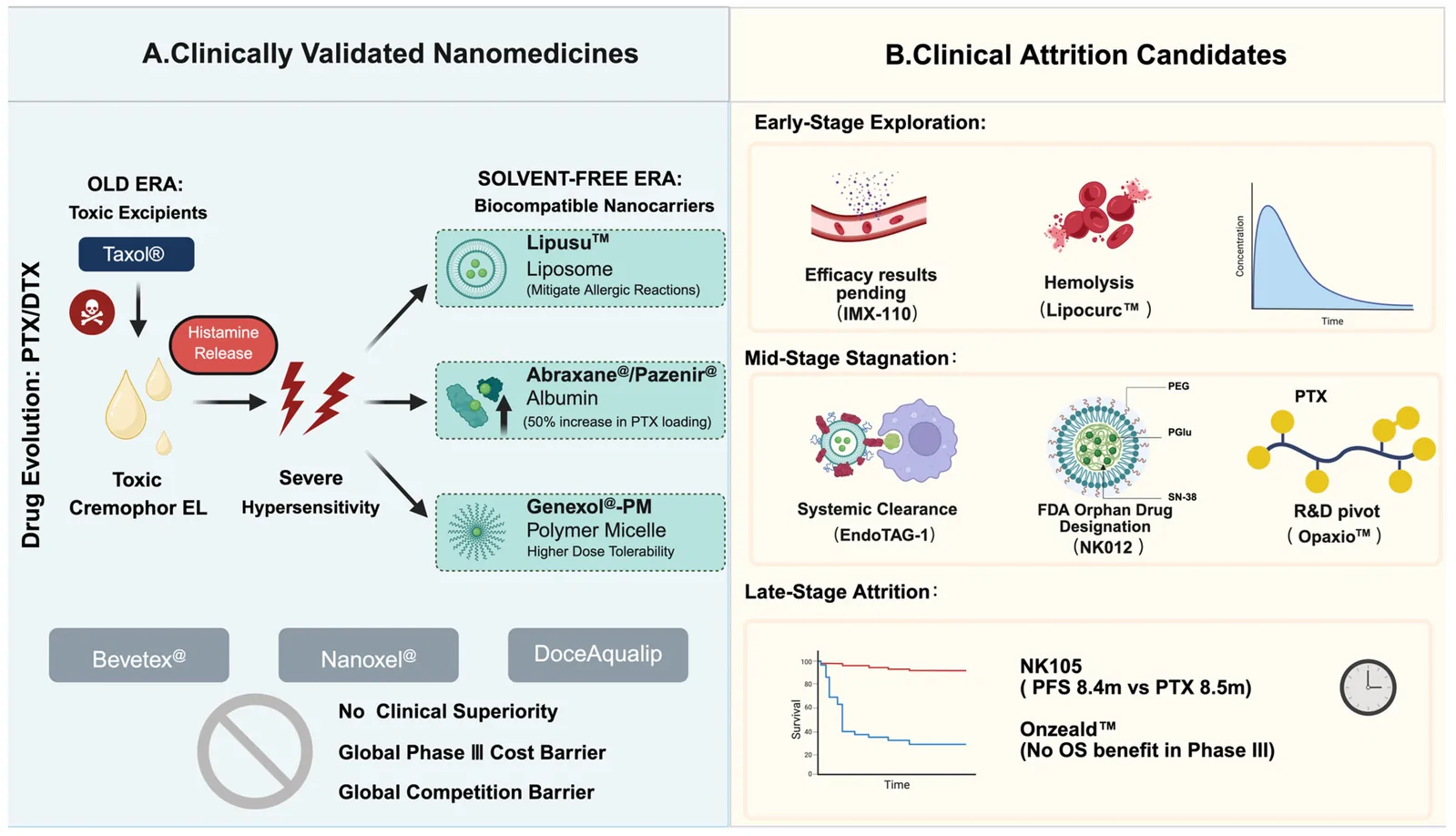
Clinical landscape of natural product-based nanomedicines in BC. (**A**) Clinically Validated Nanomedicines. The evolution of PTX/DTX formulations from toxic excipients to biocompatible, solvent-free nanocarriers (e.g., liposomes, albumin, and polymer micelles). (**B**) Clinical Attrition Candidates. Overview of nanomedicines encountering developmental hurdles, systematically categorized into three phases: early-stage exploration, mid-stage stagnation, and late-stage attrition.

Historically, the natural products that have most successfully transitioned to the clinic for BC are PTX and DTX (Figure 5A). A retrospective analysis of approved nanomedicines indicates that regulatory success heavily favors formulation simplicity and the mitigation of excipient-induced toxicity. PTX, originally isolated from the bark of the Pacific yew tree (*Taxus brevifolia*), was first commercialized as Taxol^®^. However, due to the extreme hydrophobicity of PTX, this original formulation relied on the toxic nonionic surfactant Cremophor EL (polyoxyethylene castor oil), which provoked severe histamine release and hypersensitivity reactions. To address this toxicity, solvent-free nanotechnologies emerged. Lipusu™, the world’s first approved liposomal PTX, effectively mitigated these allergic reactions by replacing the toxic solvent with biocompatible lipid bilayers [184]. Another breakthrough in solvent-free delivery is Abraxane^®^, an FDA-approved first-line treatment for metastatic BC that employs nano-albumin carrier technology. This innovation not only completely eliminated the need for premedication but also increased PTX loading by 50% and broadened its clinical indications [44]. The profound clinical success of this albumin-bound platform subsequently paved the way for generic alternatives, such as Pazenir^®^, thereby expanding global patient access [185]. Notably, Onivyde^®^ is approved for pancreatic cancer but has only completed Phase I trials in BC. Its cross-indication expansion is primarily hindered by the extremely high superiority thresholds in BC and delivery barriers driven by EPR heterogeneity and bone metastases [186].

Polymeric nanomedicines, spearheaded by Genexol^®^-PM [33,34], represent another significant milestone in solving formulation bottlenecks. Compared with Taxol^®^ and Abraxane^®^, these synthetic formulations not only allow for higher dose tolerability but also completely bypass the risks of solvent-induced hypersensitivity and potential biological contamination associated with human albumin carriers. Following this trajectory, a wave of regional solvent-free formulations successfully secured clinical approval in India by eradicating excipient toxicities. Notable examples include Bevetex^®^ [187] and Nanoxel^®^ [188] for PTX, as well as the lipid-based DoceAqualip for DTX [189]. Yet, achieving regional approval does not guarantee global commercial viability. Despite their improved safety profiles, many of these regional formulations have failed to make a global breakthrough. This translational stall is primarily driven by a lack of significant therapeutic superiority over conventional therapies, compounded by intense global market competition and the prohibitive costs of international Phase III clinical trials.

### 7.2. Clinical Attrition Candidates

While a few nanomedicines have successfully navigated the clinical pipeline, many promising candidates encounter critical hurdles that ultimately lead to a high attrition rate. As illustrated in Figure 5B, these translational failures and developmental stagnations can be systematically categorized into three distinct phases: early-stage exploration, mid-stage stagnation, and late-stage attrition.

#### 7.2.1. Early-Stage Exploration: Candidates in Phase I/II with Unconfirmed Efficacy

This category includes nanomedicines like IMX-110 and Lipocurc™, which are in the earliest stages of clinical development (Phase I/II or lower). Their primary goal is to establish safety, tolerability, and a recommended dose. However, these formulations have failed to yield robust efficacy data in breast cancer paradigms, leaving their therapeutic potential highly ambiguous. Specifically, the definitive trial status of IMX-110 remains undisclosed, while Lipocurc™ demonstrated no discernible antitumor activity in its foundational Phase I study.

#### 7.2.2. Mid-Stage Stagnation: Discontinuation Post-Phase II

This group, including EndoTAG-1, Opaxio™, and NK-012, demonstrated preliminary signals of efficacy in Phase II trials for breast cancer. However, they failed to progress to pivotal Phase III studies or were suspended/terminated. For instance, EndoTAG-1’s Phase III trial was suspended, Opaxio’s breast cancer program was discontinued, and NK-012 showed no further development after its Phase II trial. Their setbacks are attributed to a combination of modest efficacy, translational challenges (e.g., EPR effect heterogeneity), and specific drug-related issues like instability or safety concerns.

#### 7.2.3. Late-Stage Attrition: Failure in Phase III Pivotal Trials

Other nanomedicines pass early tests but fail in large-scale Phase III trials because they do not extend patient survival better than standard chemotherapy. For example, NK105 successfully reduced nerve toxicity but had a progression-free survival (PFS) of only 8.4 months, failing to beat standard paclitaxel (8.5 months) [190]. Similarly, Onzeald^TM^ reached global Phase III trials but failed to significantly improve overall survival (OS) compared with standard care, leading to its clinical rejection [191].

Ultimately, these clinical outcomes reveal that passively relying on the EPR effect cannot overcome the dense BC stroma. However, although not yet approved for BC, Onivyde^®^ provides a successful blueprint in solid tumors. Rather than forcing through the stroma, it is engulfed by perivascular macrophages, which then gradually release the active drug to penetrate deep tumor layers. This success underscores the need for future BC nanomedicines to actively exploit the tumor microenvironment instead of merely relying on passive accumulation.

**Table 7:** Natural product-based nanomedicine approved or in clinical trials for BC.

Active Ingredient	Nanocarrier	Trade Name	Final Status	Reference
PTX	Liposome	Lipusu^TM^	Approved (NMPA, 2003)	[184]
	Albumin-bound nanoparticle	Abraxane^®^	Approved (FDA, 2005; EMA, 2008)	[44]
	Albumin-bound nanoparticle	Pazenir^®^	Approved (EMA, 2019)	[185]
	Polymeric micelle	Genexol^®^-PM	Approved (MFDS, 2007; CDSCO, 2012)	[33,34]
	Polyion complex nanoparticle	Bevetex^®^	Approved (CDSCO, 2014)	[187]
	Polymer micelle	Nanoxel^®^	Approved (CDSCO, 2007)	[188]
	Polymer–drug conjugate	Opaxio^TM^	Clinical trials (Phase II, United States, 2008)	[192]
	Micellar nanoparticle	NK105	Clinical trials (Phase III, Japan, 2012)	[190]
	Liposome	EndoTAG-1	Clinical trials (Phase II, Belgium, Ukraine, France, etc., 2014)	[193]
DTX	Lipid nanosuspension	DoceAqualip	Approved (CDSCO, 2011)	[189]
Curcumin	Liposome	Lipocurc^TM^	Clinical trials (PhaseI/II, Austria, 2014)	[194]
Irinotecan	Liposome	Onivyde^®^	Clinical trials (Phase I, United States, 2020)	[186]
	Polymer–drug conjugate	Onzeald™	Clinical trials (Phase III, United States, 2011)	[191]
SN-38(the active metabolite of irinotecan)	Polymer micelle	NK012	Clinical trials (Phase II, United States, 2009)	[195]
Polyphenol curcuminoid complex/Doxorubicin	PEG-PE polymeric micelle	IMX-110	Clinical trials (PhaseI/II, United States/Australia, 2018)	[194]

### 7.3. Major Hurdles in the Clinical Translation of Nanomedicines

Ultimately, the clinical attrition of these high-profile candidates is not merely a collection of isolated setbacks, but rather symptomatic of broader, systemic bottlenecks in nanomedicine development. To fully comprehend this formidable “valley of death”, it is imperative to dissect the major hurdles in clinical translation that universally plague the field. These overarching challenges extend far beyond basic in vivo efficacy, encompassing large-scale manufacturing, batch-to-batch reproducibility, long-term stability, pharmacokinetic discrepancies, intricate regulatory considerations, and stringent safety issues.

#### 7.3.1. Manufacturing Challenges

The fabrication of natural product-based nanomedicines faces significant scale-up and stability hurdles. Inherent batch-to-batch variations in natural product extraction, combined with the intricate synthesis of multi-component nanocarriers, make it highly difficult to maintain uniform critical quality attributes (e.g., particle size, polydispersity, and encapsulation efficiency) during industrial manufacturing. Furthermore, the intrinsic sensitivity of natural compounds to environmental factors, alongside risks of premature payload leakage and nanoparticle aggregation, severely compromises the long-term stability and shelf-life of these formulations.

#### 7.3.2. Biological Challenges

The translational gap from preclinical models to humans is primarily driven by pharmacokinetic discrepancies and safety concerns. As previously discussed, inter-patient biological variability and the protein corona effect not only result in targeting failures but also induce highly complex pharmacokinetic alterations. Specifically, the historical over-reliance on the EPR effect is notoriously heterogeneous in clinical breast cancer compared to murine xenografts. Concurrently, the rapid formation of the protein corona further accelerates non-specific clearance by the mononuclear phagocyte system (MPS). Beyond pharmacokinetics, safety remains a critical barrier. While natural products are generally biocompatible, their highly concentrated localized delivery may induce unexpected toxicity, and the long-term accumulation of synthetic nanocarriers in clearance organs (e.g., liver and spleen) raises serious concerns regarding chronic hepatotoxicity and systemic immunotoxicity.

#### 7.3.3. Regulatory Challenges

The complex, multi-component nature of nanomedicines complicates the regulatory approval process. Unlike conventional small-molecule drugs, there is a lack of globally unified, standardized regulatory frameworks and evaluation protocols specifically tailored for natural product-based nanotherapeutics. Comprehensive guidelines dictating the assessment of their complex in vivo degradation profiles, immune-related adverse events, and scale-up quality standards are still evolving. Consequently, this regulatory ambiguity significantly increases the time, cost, and risk of failure during clinical development.

## 8. Conclusions and Perspectives

BC, as a malignant tumor that poses a severe threat to women’s health worldwide, has consistently motivated the pursuit of novel therapeutic strategies. Natural products have demonstrated remarkable anticancer potential owing to their multi-targeted mechanisms, which include inducing apoptosis, arresting the cell cycle, inhibiting angiogenesis and metastasis, and modulating autophagy. However, their clinical translation has long been hampered by inherent physicochemical limitations, such as poor aqueous solubility, chemical instability, and inadequate bioavailability. In recent years, the rapid development of nanodrug delivery systems (NDDS) has effectively overcome these pharmacokinetic barriers. By employing diverse targeting strategies, including passive targeting, active targeting, and stimuli-responsive targeting, NDDS have laid a solid foundation for the clinical application of natural products.

Given the profound heterogeneity of breast cancer, its various subtypes exhibit striking disparities not only in receptor expression profiles but also in molecular driving mechanisms, clinical bottlenecks, and tumor microenvironment (TME) characteristics. Consequently, tailoring nanoformulations to the distinct molecular phenotypes of each subtype has emerged as a core strategy for achieving precise delivery and personalized intervention of natural products.

Despite its great promise, this field still faces formidable challenges on the path to clinical translation, including insufficient delivery and transfection efficiencies, potential carrier-related toxicity, and difficulties in large-scale production. First, to fully unlock the clinical potential of natural product nanoformulations, efforts can be made through a multi-pronged approach that optimizes carrier physicochemical properties, remodels the tumor microenvironment, incorporates external physical field assistance, and applies biomimetic delivery technologies to break through the cascading biological barriers. Second, to address the issues of complex multi-component synthesis and substantial batch-to-batch variability, it is advisable to vigorously develop self-assembly technologies for natural products (i.e., pure-drug nanoparticles) or to employ a single multifunctional natural coating. By simplifying the formulation from the outset and reducing reliance on inert synthetic carriers, the difficulty of industrial scale-up can be substantially lowered. Finally, given the complexity of natural product nanomedicines, dedicated in vitro release and degradation testing methods should be established that can realistically predict their in vivo pharmacokinetic behavior, thereby providing core data to persuade regulatory agencies.

In summary, the deep integration of natural products with nanodelivery technologies has established a novel therapeutic paradigm for targeted intervention in breast cancer. In the future, with the continuous iteration of intelligent nanoplatforms and the progressive refinement of standardized evaluation systems, natural product-based nanomedicines will undoubtedly play a pivotal role in precision treatment and combination therapy for breast cancer, ultimately benefiting a broad patient population.

## Figures and Tables

**Table 1:** Summary of the anti-breast cancer mechanisms mediated by natural products.

Category	Representative Natural Products	Main Source	Main Mechanisms	Ref
Flavonoids	Quercetin	Onion, kale, apple, broccoli	Induces cell cycle arrest Induces apoptosis Inhibits tumor metastasis Regulates angiogenesis	[11]
	Luteolin	Honeysuckle, chrysanthemum, Brussels sprouts, broccoli, peppers, wine	Induces apoptosis Inhibits tumor metastasis Inhibits angiogenesis Induces cell cycle arrest	[12,13]
	Puerarin	Kudzu plant (Radix puerariae)	Induces apoptosis Inhibits proliferation and metastasis	[14]
	Apigenin	Onions, grapefruit, parsley	Induces apoptosis and cell cycle arrest Inhibits proliferation and oxidative stress	[15,16]
	Isoliquiritigenin	*Glycyrrhizae Rhizoma*	Induces apoptosis and cell cycle arrest Inhibits tumor angiogenesis Inhibits proliferation and metastasis	[17]
	Curcumin	Turmeric	Induces apoptosis Inhibits angiogenesis and proliferation	[18]
Polyphenols and flavonoids	Epigallocatechin gallate	Tea	Induces apoptosis Inhibits proliferation and metastasis	[19]
	Resveratrol	Peanuts, berries, grapes, Japanese knotweed, fungi, red wine	Induces apoptosis and autophagy Inhibits proliferation	[20]
	Cyanidin-3-glucoside	Flowers, fruits	Induces cell cycle arrest Inhibits proliferation and metastasis	[21]
	Kaempferol	Tea, broccoli, witch hazelnut, propolis, grapefruit	Induces apoptosis and cell cycle arrest Inhibits tumor metastasis	[22]
Alkaloids	Evodiamine	Evodiae Fructus plants	Induces cell cycle arrest Induces apoptosis Inhibits proliferation and metastasis	[23]
	Camptothecin	Camptotheca acuminita	Induces apoptosis and DNA damage	[24]
	Berberine	Coptis chinensis	Induces autophagy, apoptosis, cell cycle arrest, and DNA damage Inhibits proliferation and metastasis	[25]
	Piperlongumine	Piper longum	Induces apoptosis, autophagy, and cell cycle arrest Inhibits metastasis	[26]
Others	Triptolide and tripterine	Tripterygium wilfordi	Induces cell cycle arrest Inhibits metastasis and angiogenesis	[27]
	Ginsenosides	Ginseng root	Induces apoptosis Inhibits proliferation	[28]

**Table 2:** Characteristics and translational prospects of major nanocarriers for BC drug delivery.

Nanocarrier	Key Advantages	Primary Limitations	Translational Potential
Liposomes	High biocompatibility and biodegradability; amphiphilic structure for co-delivery and high loading; easily functionalized for active targeting.	Prone to lipid oxidation and payload leakage; rapid mononuclear phagocyte system clearance; cationic lipid toxicity.	High FDA-approved precedents (Doxil) exist and are excellent for translating multi-component herbal extracts.
Solid Lipid Nanoparticles	High physical stability and good bioavailability; excellent controlled release profile; scalable and solvent-free production.	Low loading capacity for hydrophilic compounds; risk of drug expulsion during storage; complex Colloidal State.	Moderate to High The scale-up advantages are notable, yet the clinical pipeline remains at a relatively early stage.
Polymeric Micelles	Core–shell structure ideally solubilizes highly hydrophobic natural products; small size (~50 nm) enables deep tumor penetration; highly versatile chemical composition.	Risk of premature dissociation in systemic circulation and carrier degradation.	Moderate Precedents exist (Genexol^®^-PM), and it requires stimuli-responsive cross-linking for optimal stability.
Dendrimers	Highly branched, monodisperse architecture and numerous peripheral groups for multi-drug conjugation and targeting.	Step-by-step synthesis is tedious and costly, and cationic surfaces can induce significant cytotoxicity and hemolysis.	Low to Moderate Significant regulatory hurdles due to synthetic complexity and safety concerns.
Metal NPs	Enables theranostic applications (imaging + therapy); precise drug loading with rigid scaffolds; facile surface functionalization via Au-S chemistry.	Long-term accumulation toxicity; lack of biodegradability; difficult systemic clearance; potential immunogenicity.	Low More suitable for localized injection or highly specific theranostic applications rather than systemic administration.
Protein Nanocarriers	Exceptional biocompatibility; inherent multifunctional binding sites; inherent tumor accumulation and transcytosis capability.	High production costs; potential protein denaturation during payload encapsulation and batch-to-batch consistency challenges.	High Albumin-based carriers have a proven track record in breast cancer (Abraxane^®^) and high clinical acceptance.

## Data Availability

No new data were created or analyzed in this study. Data sharing is not applicable to this article.

## Abbreviations

The following abbreviations are used in this manuscript:

ACP	Amorphous calcium phosphate
BA	Boronopicolinic acid
BC	Breast cancer
BCSCs	Breast cancer stem cells
BRCA	Breast Cancer susceptibility gene
CDK	Cyclin-dependent kinase
CDSCO	Central Drug Standards Control Organization of India
CPT	Camptothecin
DAV	Drug Administration of Vietnam
DTX	Docetaxel
EGCG	Epigallocatechin gallate
EGFR	Epidermal growth factor receptor
EMA	European Medicines Agency
EMT	Epithelial–mesenchymal transition
EPR	Enhanced permeability and retention
ER	Estrogen receptor
FA	Folic acid
FDA	Food and Drug Administration
GA	Gallic acid
GOQD	Graphene oxide quantum dots
GSH	Glutathione
Gem	Gemcitabine
HA	Hyaluronic acid
HER2	Human epidermal growth factor receptor 2
HR	Hormone receptor
HSP 70	Heat shock protein 70
IFP	Interstitial fluid pressure
L-MSNs	Lipid-coated mesoporous silica nanoparticles
LCST	Lower critical solution temperature
LHRH	Luteinizing hormone-releasing hormone
MFDS	Ministry of Food and Drug Safety
MIONPs	Magnetic iron oxide nanoparticles
MMP-2	Matrix metallopeptidase 2
MMP-9	Matrix metallopeptidase 9
MOF	Modified metal–organic framework
MSNs	Mesoporous silica nanoparticles
NP	Nanoparticle
NDDS	Nanoparticle-based delivery systems
NIR	Near-infrared
NMPA	National Medical Products Administration
NRP-1	Neuropilin-1
PARP	Poly(ADP-ribose) polymerase
PCMs	Phase change materials
PD-1	Programmed cell death 1
PD-L1	Programmed death-ligand 1
PDH	Philippines: Department of Health
PDT	Photodynamic therapy
PEG	Polyethylene glycol
PFP	Perfluoropentane
P-gp	P-glycoprotein
PL	Piperonamide
PLL	PEGylated poly-L-lysine
PNIPAM	Poly(N-isopropylacrylamide)
PPA	Pyro-de-magnesium chlorophyll-a
PPRM	Pyropheophorbide-a (Ppa) micelle
PPY	Polypyrrole
PR	Progesterone receptor
PS	Photosensitizers
PTT	Photothermal therapy
PTX	Paclitaxel
RFMPH	Russian Federation: Ministry of Public Health
RGD	Arg-Gly-Asp
ROS	Reactive oxygen species
SA	Sialic acid
SDT	Sonodynamic therapy
SELEX	Systematic evolution of ligands by exponential enrichment
SLNs	Solid lipid nanoparticles
SPIONs	Superparamagnetic iron oxides
THZ	Thioridazine
TME	Tumor microenvironment
TNBC	Triple-negative breast cancer
TNF	Tumor necrosis factor
Tf	Transferrin
Trop-2	Trophoblast cell-surface antigen 2
VEGF	Vascular endothelial growth factor
ZIF-8	Zeolitic imidazolate framework-8
shRNA	small hairpin RNA
